# Thermophilic methane oxidation is widespread in Aotearoa-New Zealand geothermal fields

**DOI:** 10.3389/fmicb.2023.1253773

**Published:** 2023-08-31

**Authors:** Karen M. Houghton, Carlo R. Carere, Matthew B. Stott, Ian R. McDonald

**Affiliations:** ^1^Te Pū Ao | GNS Science, Wairakei Research Centre, Taupō, New Zealand; ^2^Te Aka Mātuatua | School of Science, Te Whare Wānanga o Waikato | University of Waikato, Hamilton, New Zealand; ^3^Te Tari Pūhanga Tukanga Matū | Department of Chemical and Process Engineering, Te Whare Wānanga o Waitaha | University of Canterbury, Christchurch, New Zealand; ^4^Te Kura Pūtaiao Koiora | School of Biological Sciences, Te Whare Wānanga o Waitaha | University of Canterbury, Christchurch, New Zealand

**Keywords:** Aotearoa-New Zealand, geothermal, methane, methanotroph, oxidation, thermophile, transcriptomics

## Abstract

Geothermal areas represent substantial point sources for greenhouse gas emissions such as methane. While it is known that methanotrophic microorganisms act as a biofilter, decreasing the efflux of methane in most soils to the atmosphere, the diversity and the extent to which methane is consumed by thermophilic microorganisms in geothermal ecosystems has not been widely explored. To determine the extent of biologically mediated methane oxidation at elevated temperatures, we set up 57 microcosms using soils from 14 Aotearoa-New Zealand geothermal fields and show that moderately thermophilic (>40°C) and thermophilic (>60°C) methane oxidation is common across the region. Methane oxidation was detected in 54% (*n* = 31) of the geothermal soil microcosms tested at temperatures up to 75°C (pH 1.5–8.1), with oxidation rates ranging from 0.5 to 17.4 μmol g^−1^ d^−1^ wet weight. The abundance of known aerobic methanotrophs (up to 60.7% *Methylacidiphilum* and 11.2% *Methylothermus*) and putative anaerobic methanotrophs (up to 76.7% Bathyarchaeota) provides some explanation for the rapid rates of methane oxidation observed in microcosms. However, not all methane oxidation was attributable to known taxa; in some methane-consuming microcosms we detected methanotroph taxa in conditions outside of their known temperature range for growth, and in other examples, we observed methane oxidation in the absence of known methanotrophs through 16S rRNA gene sequencing. Both of these observations suggest unidentified methane oxidizing microorganisms or undescribed methanotrophic syntrophic associations may also be present. Subsequent enrichment cultures from microcosms yielded communities not predicted by the original diversity studies and showed rates inconsistent with microcosms (≤24.5 μmol d^−1^), highlighting difficulties in culturing representative thermophilic methanotrophs. Finally, to determine the active methane oxidation processes, we attempted to elucidate metabolic pathways from two enrichment cultures actively oxidizing methane using metatranscriptomics. The most highly expressed genes in both enrichments (methane monooxygenases, methanol dehydrogenases and PqqA precursor peptides) were related to methanotrophs from Methylococcaceae, Methylocystaceae and Methylothermaceae. This is the first example of using metatranscriptomics to investigate methanotrophs from geothermal environments and gives insight into the metabolic pathways involved in thermophilic methanotrophy.

## 1. Introduction

The greenhouse gas methane (CH_4_) is responsible for a large proportion of global climate change, being 28 times more effective than carbon dioxide (CO_2_) at absorbing infra-red radiation (Forster et al., 2021). The global methane budget is ~730 Tg yr^−1^ from both natural and anthropogenic sources; with ~5% of this methane consumed by methanotrophic bacteria before it reaches the atmosphere (Canadell et al., 2021). It is therefore important to understand the distribution and diversity of methanotrophic bacteria to gain insight into the ecological processes that modulate methane emissions and its oxidation.

Aerobic methanotrophic bacteria have been detected and/or isolated from nearly all known habitats associated with methane emissions; including wetlands, rice paddy fields, landfills, and geothermal areas (Tsubota et al., 2005; Geymonat et al., 2011; Danilova et al., 2013; Henneberger et al., 2014). While knowledge concerning methanotroph ecology within many of these ecosystems is extensive (Hanson and Hanson, 1996; Jiang et al., 2010), the understanding of the distribution and diversity of methane oxidizing bacteria in geothermal fields remains limited (Houghton et al., 2019). Geothermal methane emissions from magma degassing amounts to ≤71 Tg yr^−1^ (Canadell et al., 2021), but geothermal and volcanic areas are inhospitable for most known species of methanotrophs due to acidic pH, hot temperatures and low oxygen concentrations (Op den Camp et al., 2009). Evidence of methanotrophic activity in geothermal areas was first reported in 2005 (Castaldi and Tedesco, 2005), and a number of thermophilic aerobic methanotrophs have been isolated from geothermal soils, including the description of three genera (*Methylacidiphilum*, *Methylacidimicrobium*, *Candidatus* Methylacidithermus) within the phylum Verrucomicrobiota (van Teeseling et al., 2014; Picone et al., 2021a). Thermophilic methanotrophs from these areas demonstrate metabolic flexibility, using or consuming a wide range of energy sources in addition to methane, including hydrogen (Carere et al., 2017), hydrogen sulfide (Schmitz et al., 2023), methanethiol (Schmitz et al., 2022) and short-chain alkanes (Awala et al., 2021). However, little is known about the phylogenetic diversity of thermophilic methanotrophs from geothermal areas, perhaps due to the difficulty of culturing methane-oxidisers, from a failure to replicate environmental conditions *in vitro*, or because there are biological restrictions on methane oxidation at hotter temperatures, such as methane solubility or enzyme instability (Houghton et al., 2019).

Molecular techniques have frequently been used to detect methanotrophs within environmental samples. These methods commonly include DNA amplicon sequencing of the 16S rRNA gene (Bodrossy et al., 1997), or functional genes associated with methanotrophy such as methane monooxygenase (McDonald et al., 1995; Sharp et al., 2012; Gagliano et al., 2014), and metagenomics (Håvelsrud et al., 2011; Nguyen et al., 2018; Taubert et al., 2019). However, DNA sequencing techniques provide little information about the activity of microbial community members, gene expression or responses to environmental conditions (Wang et al., 2012). Considering up to 80% of soil microorganisms are believed to exist in a state of dormancy (Lennon and Jones, 2011), DNA sequencing of these microorganisms can obscure assessments of metabolic activity within an environment. Previous studies targeting methanotrophs have used transcriptomics within hydrothermal vents (Lesniewski et al., 2012; Olins et al., 2017), peatlands (Liebner and Svenning, 2013; Esson et al., 2016) and landfill soil (Henneberger et al., 2014), but not geothermal soil or hot spring ecosystems.

**Figure 1:**
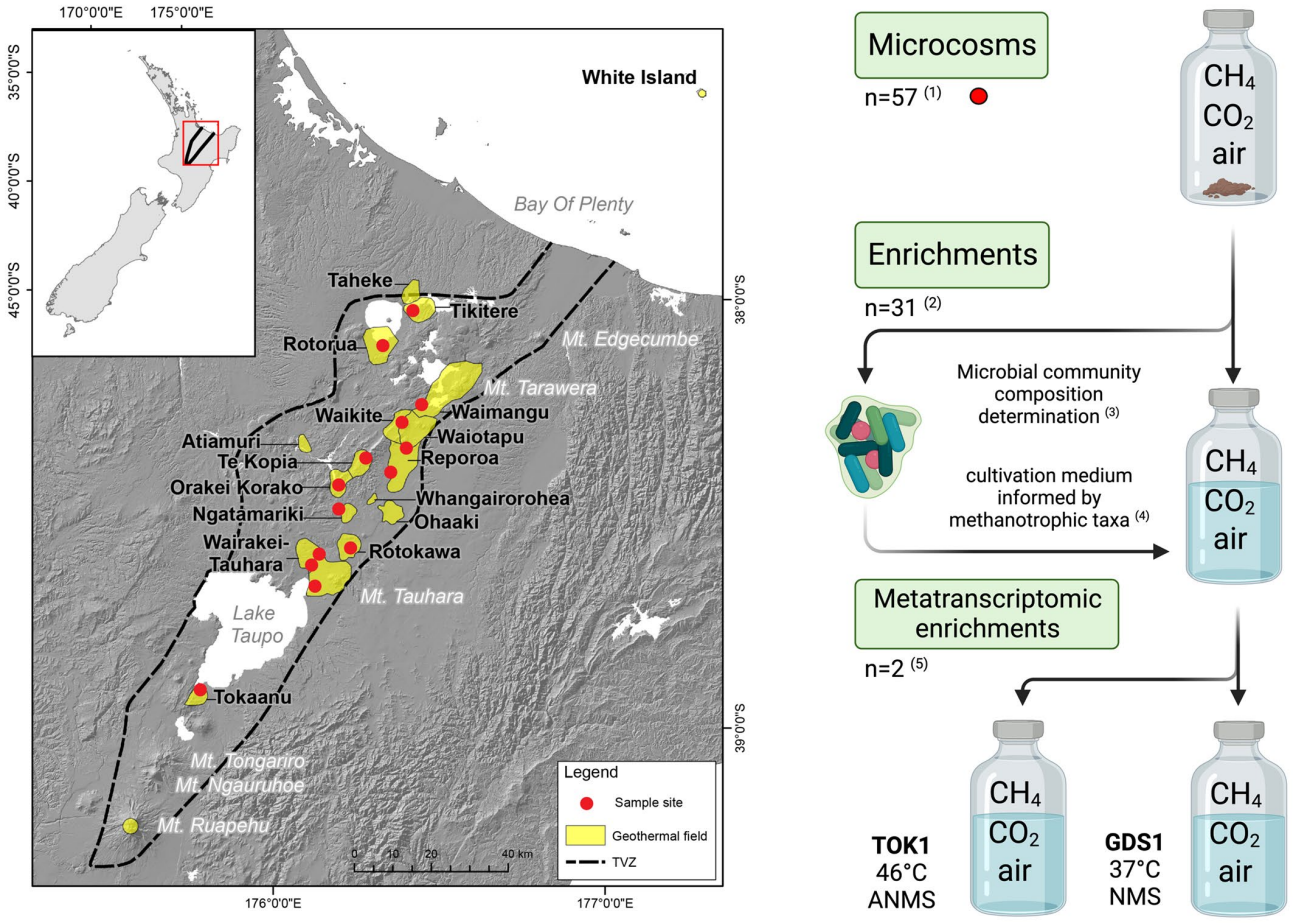
Map of the Taupō Volcanic Zone (TVZ), Aotearoa-New Zealand, and a schematic of the experimental study for this research. A total of 57 samples were collected (red circles) from 14 geothermal fields. (1) Microcosms were prepared using a single soil (*n* = 43), sediment (*n* = 11) or water/biofilm (*n* = 3) sample with a headspace of (v/v) 10% CH_4_, 1.3% CO_2_ in air. (2, 3) The microbial community composition of the 31 microcosms that showed positive CH_4_ oxidation were determined by 16S rRNA gene amplicon sequencing. (4) Enrichment cultures using a subsample of the microcosm substrate were inoculated into NMS media and other media informed by methanotrophic taxa detected in the microcosm microbial communities. (5) Two enrichments, TOK1 (Tokaanu) and GDS1 (Golden Springs), showing strong CH_4_ consumption were further subcultured into ANMS (46°C) and NMS (37°C) media, respectively, for metatranscriptomic analyses. Individual microcosm and enrichment cultivations were undertaken as close as possible to their measured *in situ* temperatures (see Table 1). Map adapted from Power et al. (2018), and schematic created in www.biorender.com/.

In this study, we combined the use of 16S rRNA gene amplicon sequencing on geothermal samples from throughout the Taupō Volcanic Zone (TVZ; Aotearoa-New Zealand) with the quantification of methane oxidation rates to detect the presence and activity of thermophilic and thermotolerant methanotrophs. The TVZ spans 6,000 km^2^ of the central North Island of Aotearoa-New Zealand and extends from the active volcanoes of Whakaari/White Island to Mount Ruapehu (Figure 1). The area contains more than 20 geothermal fields that are characterized by an abundance of hot springs, geysers, and mud pools (Giggenbach, 1995). Prior analysis of gases emitted from geothermal fields within the TVZ has found that most systems release 0.1 to 5.0% CH_4_ (v/v), with some sites, such as Golden Springs, emitting up to 19.3% CH_4_ (v/v) (Giggenbach, 1995).

To determine thermophilic methanotrophic activity in these geothermal areas, we emended soil and sediment microcosms with various media designed to promote methanotrophic growth. Methane oxidation in each microcosm was quantified along with microbial community composition. Two enrichments were then selected which showed both elevated rates of methane oxidation and abundant methanotroph communities. Metatranscriptomic analyses of the two enrichments were used to assess potential methanotrophic metabolic activity.

## 2. Materials and methods

An overview of the experimental workflow is shown in Figure 1.

### 2.1. Microcosms

#### 2.1.1. Sampling location overview and characterization

Microcosm samples were collected from geothermally heated soils (with surface temperatures >55°C), hot spring waters, sediments or associated biofilms from 14 geothermal fields within the TVZ (Figure 1; Table 1). Site selection was partially informed from the 1,000 Springs Project dataset (Power et al., 2018) by screening for sites with either high *in situ* concentrations of methane, or with a high proportion of 16S rRNA gene sequences assigned as putative methanotrophs. Individual physicochemical observations for these sites and associated microcosms are listed in Supplementary Table 1.

**Table 1:** Physicochemical characteristics, number of microcosms, and location of the 14 geothermal systems from the Taupō Volcanic Zone (TVZ), Aotearoa-New Zealand sampled for this study.

Geothermal field.	Nomenclature	Numbers of microcosms	*In situ* temperature range (°C)	pH	Maximum *in situ* CH_4_ in geothermal field (ppmv)
Craters of the Moon	COM	2	70.2–76.0	6.4–7.6	n.d.
Golden Springs	GDS	2	38.0–39.9	7.0–7.2	n.d.
Loop Road	LPR	4	50.0–85.0	1.5–5.4	3896.2
Ngatamariki	NGM	3	51.0–70.0	3.0–7.0	n.d.
Orakei Korako	OKO	1	71.6	4.3	5.9
Rotokawa	RTK	1	77.3	2.1	n.d.
Te Kopia	TKA	10	51.1–85.3	4.2–5.3	98.6
Tikitere	TKT	2	35.0–75.8	2.6–3.0	13.8
Tokaanu	TOK	13	62.5–82.5	5.4–9.2	21.3
Waimangu	WAM	1	73.0	4.6	n.d.
Waipahihi	WAP	1	45.0	6.7	n.d.
Whakarewarewa Village	WHV	8	55.7–84.5	2.5–8.7	112
Waikite Valley	WKT	5	39.7–72.4	4.0–8.3	732.5
Wairakei Thermal Valley	WTV	3	65.3–79.5	3.7–7.7	1.8

*In situ* temperature and pH measurements detail the range of measured conditions for individual samples collected for microcosm studies (Supplementary Table 1). n.d., not determined.

Ground temperatures were measured using a 51 II thermal probe (Fluke) inserted into the soil or sediment at 20 cm depth. Soil gas samples were collected, where possible, using a custom-built stainless steel sampling tube inserted into the soil to the same depth. Before collecting the gas sample, and to avoid atmospheric contamination, 700 ml of gas (>3× tube volume) was drawn through the sampling tube and discarded. 25 ml of gas was then slowly extracted from the soil and injected through a gas-tight three-way valve into a pre-evacuated ‘Air and Gas Sampling Bag’ (Calibrated Instruments Inc). Gas samples were processed within 24 h on a Peak Performer 1 Gas Analyzer (Peak Laboratories LIC) equipped with a flame ionization detector (FID) and a Unibeads 60/80 column. Soil/sediment samples (~2 g) were collected using a trowel and spatula previously sterilized with ethanol and were immediately transferred to pre-autoclaved serum vials (Wheaton Industries) which were then capped with sterile butyl rubber stoppers and sealed with aluminum crimps. A separate duplicate soil/sediment sample was taken for pH measurement by suspending ~1 g of soil in 10 mL RO H_2_O at room temperature (Table 1; Supplementary Table 1).

#### 2.1.2. Methane oxidation by microcosms

A gas mixture (v/v), consisting of 80% CH_4_, 15% CO_2_ and 5% O_2_ (14.3 mL), was injected into the serum vial headspace through a sterile filter resulting in final gas headspace concentrations of 10% CH_4_, 1.3% CO_2_ and 21.6% O_2_ of each microcosm (Supplementary Table 1). Control vials with no soil were set up with the same gas headspace. Vials were incubated at temperatures (46, 50, 60, 68, 70, 75 or 80°C) approximating the closest *in situ* sample temperature (controls incubated at 60°C). Microcosms from Golden Springs (GDS) were incubated at 50°C to target thermophilic methanotrophs, although the *in situ* temperature was 38°C. At 3–4 day intervals, vials were moved to a 20°C water bath and equilibrated to room temperature (20°C) for 5 h, to ensure accurate comparisons of pressure and moles of methane consumed Headspace gas compositions were then quantified by GC-FID by removing 500 μL with a gastight syringe (SGE Analytical Science) and diluting to 5 mL with air.

Methane oxidation rates (μmol g^−1^ d^−1^ wet weight) were calculated using linear regression. If microcosms showed a greater loss of methane than controls (following sampling without replacement) for five consecutive readings, and if the coefficient of determination of linear regression (R^2^) was >0.5, microcosms were designated as positive for methane oxidation. Supplementary Figure 1 shows methane oxidation rate graphs for selected soil microcosms. After positive oxidation was determined, or following at least 4 weeks of headspace measurements for microcosms that did not oxidize methane, DNA was extracted from all microcosms.

#### 2.1.3. Microcosm DNA extraction and sequencing

DNA was extracted from soil microcosms using a modified protocol for the NucleoSpin Soil kit (Macherey-Nagel) (Houghton and Stewart, 2019). DNA was amplified using universal primers for the V4 region (515F, 806R) of the 16S rRNA gene (Caporaso et al., 2011). PCR was carried out in 50 μL reaction volumes containing 100 μM dNTPs, 0.5 μM primers, 1 U i-Taq (iNtRON Biotechnology) and 7 μL of an enhancer solution (2.7 M betaine, 0.2 M trehalose, 6.7 mM DTT, 0.06 mg ml^−1^ BSA and 0.07% DMSO). The final concentration of MgCl_2_ was 1.5 mM. DNA templates from microcosms were used at final concentrations of 10–50 ng reaction^−1^. Three PCR amplicons (~300 bp) for each sample were pooled. The amplicons were then purified using the NucleoSpin Gel and PCR Clean-up kit (Macherey-Nagel) and Agencourt AmPure XP (Beckman Coulter). Amplicon libraries using the PCR products were prepared and sequenced by Macrogen Inc. Sequencing data was deposited in the NCBI BioProject database (PRJNA766707 and PRJNA546003).

#### 2.1.4. Community 16S rRNA gene sequence processing and diversity metric assessment

The quality of raw read data was assessed using FastQC (RRID:SCR_014583) (Andrews, 2010). Paired-end sequence reads were merged and filtered using USEARCH v7.0, with a maximum expected error of 1 (Edgar, 2010). Remaining sequences either >500 bp (to remove poor quality sequences at the end of long reads) or <200 bp (the minimum required for taxonomic classification) were removed using mothur (RRID:SCR_011947) v1.35.1 (Schloss et al., 2009). A *de novo* database of ≥97% similar sequence Operational Taxonomic Units (OTUs) was created in USEARCH (Edgar, 2010). Raw sequences were mapped against this *de novo* database to generate counts of sequences matching OTUs (i.e., taxa) for each sample. Using QIIME (RRID:SCR_008249) v1.9.1 (Caporaso et al., 2010), taxonomy was assigned to each OTU by using the RDP classifier v2.2 (Wang et al., 2007) with a confidence threshold of 0.5 and trained on the SILVA (RRID:SCR_006423) 16S rRNA gene database (version 123) (Quast et al., 2013). Chloroplast and mitochondrial OTUs were removed and all samples were rarefied to the lowest sample read count (n = 149,400).

All OTUs identified as belonging to the methanotrophic families Beijerinckiaceae or Methylocystaceae (Alphaproteobacteria); the order Methylococcales (Gammaproteobacteria); or as Methylacidiphilum (Verrucomicrobiota) were manually checked against the NCBI Nucleotide (RRID:SCR_004860) database using a discontiguous megablast (Altschul et al., 1990). OTUs that were > 90% related to a described methanotrophic species were identified as putative methanotrophs. No OTUs were identified from the NC10/Methylomirabilis phylum were identified.

The core diversity workflow within QIIME (Caporaso et al., 2010) was used to analyze sequencing data, by creating multiple rarefactions of the data, calculating and comparing alpha and beta diversity of the samples, and summarizing taxa across all samples and as a function of methane oxidation status. Chao1 [a non-parametric estimate of species richness (Chao, 1984)] and the Shannon (Shannon and Weaver, 1963) and Simpson (Simpson, 1949) indices (quantitative measures of diversity and abundance) were used as alpha diversity metrics. A non-parametric two-sample t-test on the Shannon and Simson indices was performed using QIIME. To assess significant differences between genera, OTUs from all microcosms were filtered to remove unclassified sequences, and Kruskal-Wallis tests (McDonald, 2014) performed on genus clusters.

### 2.2. Methanotroph enrichments

All microcosms displaying methane oxidation were selected for enrichment of methanotrophs using a variety of media (Supplementary Table 2; Supplementary Material 1). Each microcosm was inoculated into a modified Nitrate Mineral Salts medium (mNMS), which was designed to be a non-specific methanotroph medium. In addition to mNMS, soil from each microcosm with positive methane consumption was also inoculated into at least one other growth medium. The additional medium was selected after consideration of the soil pH and putative methanotrophs detected in the microcosm communities (see Supplementary Table 2; Supplementary Material 1 for further details). All media were adjusted to reflect the pH of the starting microcosm source material.

Between 0.3–0.5 g of the soil or sediment sample from selected microcosms (Supplementary Table 2) were aseptically transferred to between two to four serum bottles each containing 40 mL of a different sterile medium for enrichment. The final gas headspace composition (v/v) was ~10% CH_4_, 1% CO_2_ and 22% O_2_ (balance N_2_). Enrichment serum vials were incubated at temperatures reflecting *in situ* sample temperature (37, 46, 50, 60, 70, or 75°C), with shaking at 150 r.p.m. Every 3–4 days, gas compositions were measured as previously above. Rates of methane oxidation (μmol d^−1^) were calculated using linear regression. Supplementary Figure 2 shows methane oxidation rate graphs for selected enrichments. For those enrichments that did not oxidize all methane within the headspace, a coefficient of determination (*R*^2^) greater than 0.5 was designated as positive for methane oxidation.

### 2.3. Metatranscriptome enrichments

#### 2.3.1. Culture conditions for RNA extraction

Two methane-oxidizing geothermal enrichment cultures (GDS1 and TOK7) were selected for metatranscriptome sequencing Supplementary Material 1 Supplementary methods, Supplementary Table 3) based upon the relative abundances of methanotroph-associated 16S rRNA gene sequences and measured methane consumption rates. New enrichment cultures were prepared by inoculating the initial GDS1 and TOK7 enrichment cultures into fresh media in triplicate. Cultures were prepared in 114 mL serum vials with 30 mL of the relevant medium. Each sealed vial contained an air headspace supplemented with (v/v) ~ 10% CH_4_ and ~1% CO_2._ GDS1 enrichments were incubated at 37°C, the optimum growth temperature for *Methylococcus* spp. (Bowman et al., 1993), while TOK7 enrichments were incubated at 46°C to promote growth of Methylothermaceae (Houghton et al., 2019).

The extent of methane oxidation within each metatranscriptome enrichment was assessed by measuring the headspace gas composition over a 24-h period using GC-FID as described above. RNA was stabilized by the addition of 20 mL of RNAlater (Invitrogen) following 24 h of incubation and verification of methane consumption (> 5,000 μmoles CH_4_ d^−1^). Cells were collected via centrifugation at 16,000× *g* for 15 min at 10°C, with the cell pellet resuspended in 10 mL of RNAlater.

#### 2.3.2. RNA extraction and sequencing

RNA extraction and sequencing were performed by Novogene as per (Liu et al., 2015). The cDNA library was sequenced using the NovaSeq SP platform (see Supplementary Material 1 Supplementary methods).

#### 2.3.3. Bioinformatics

cDNA sequence analyses were performed within the Galaxy (RRID:SCR_006281) web platform (Afgan et al., 2016) with all settings for the individual pipeline wrappers using default settings unless stated otherwise. Initial raw read data was assessed using FastQC (v0.69) (Andrews, 2010), with reads trimmed using Trimmomatic (RRID:SCR_011848) (v0.36.2) (Bolger et al., 2014). The average quality required was set to a Phred score of 25 for GDS samples, and 20 for TOK samples, based on their FastQC reports. Reads with a length < 120 bp after trimming were removed. Forward and reverse reads for each sample were concatenated into two files using cat (v.0.1.0) (Gruening, 2014) and then assembled into contigs using Trinity (RRID:SCR_013048) (v2.2.0) (Grabherr et al., 2011).

Predicted genes within the contigs were identified using MetaGeneMark (v3.25) (Besemer and Borodovsky, 1999; Zhu et al., 2010). Identical sequences were removed using the standalone Java applet, DuplicatesFinder (Khan, 2009). The remaining sequences were clustered using the program CD-HIT (RRID:SCR_007105) (Huang et al., 2010) with sequences clustered at 90% nucleotide sequence identity for the GDS1 sample and at 85% nucleotide sequence identity for the TOK7 sample. One representative sequence from each cluster identified by cd-hit-est was retained for further analysis. The predicted genes were analyzed using BLASTX (RRID:SCR_001653) (Altschul et al., 1997) on a downloaded BLAST+ executable (v2.7.1) (Camacho et al., 2009) and a local copy of the non-redundant (nr) protein sequences (NCBI - 19/02/2018). Predicted proteins from the GDS1 and TOK7 samples were manually searched for modules within the “Methane Metabolism,” and “Nitrogen Metabolism” pathways in the KEGG (RRID:SCR_012773) database (Kanehisa et al., 2016). Finally, transcripts from the trimmed, non-concatenated datasets were quantified against the predicted genes from the Trinity assemblies using Salmon (RRID:SCR_017036) (0.8.2) (Patro et al., 2017), and the most highly expressed transcripts, based on Transcripts Per Million (TPM) across all three biological replicates, were identified for each sample.

## 3. Results and discussion

### 3.1. Rapid methane oxidation observed within thermophilic microcosms

**Figure 2:**
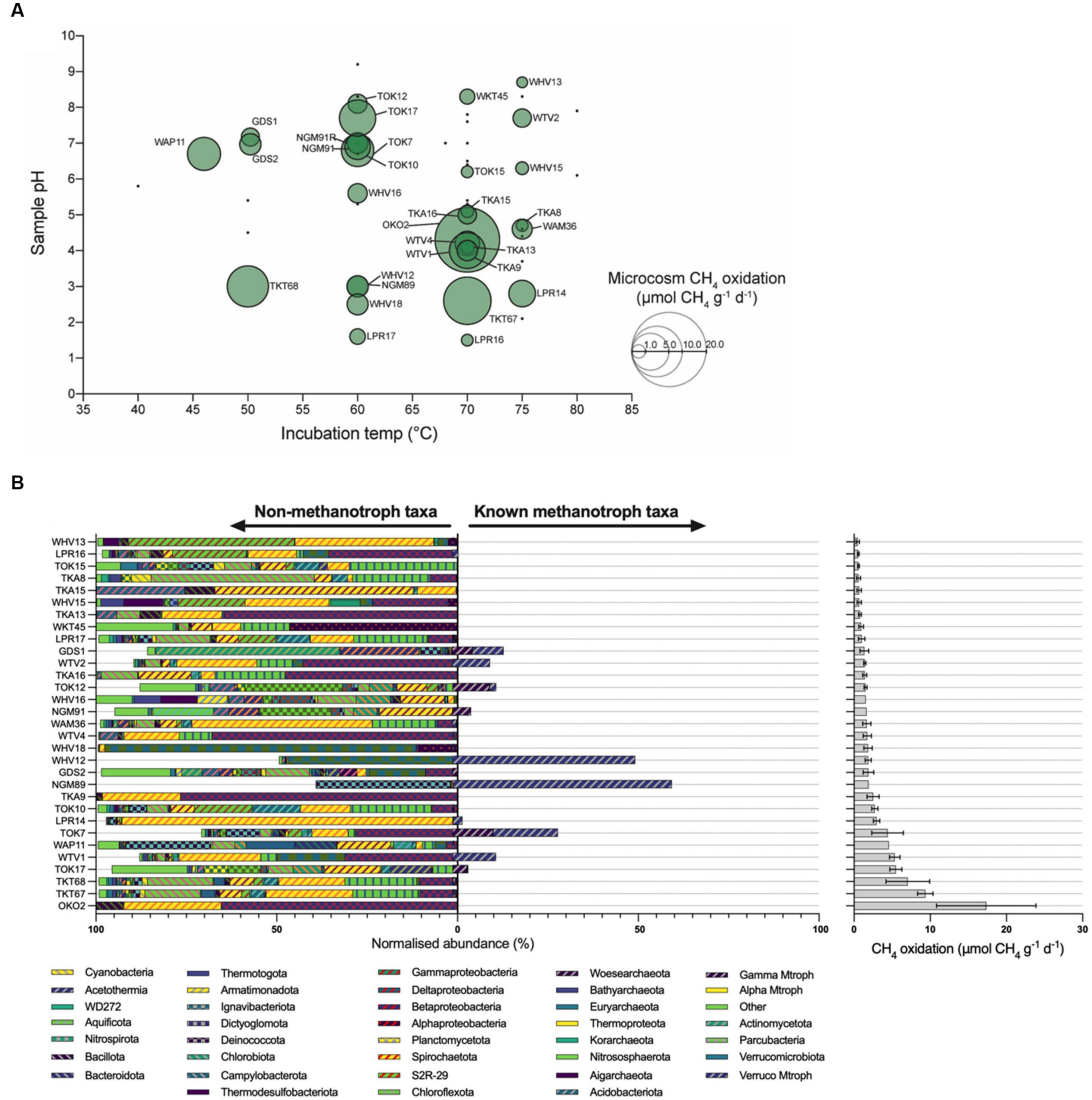
Microcosm methane oxidation rates and microbial diversity. **(A)** Bubble charts illustrating observed methane oxidation rates of soil microcosms and as a function of temperature and pH. Green circles represent samples that oxidized methane and are labeled with an identifier. Bubble size is proportional to the rate of observed methane oxidation. Black dots denote samples that did not oxidize methane. **(B)** Bar chart of phyla identified in 16S rRNA gene sequencing of microcosms, with microcosm methane oxidation rates (μmol CH4 g^−1^ d^−1^). Microcosm communities suggest unknown methanotrophs may be responsible for observed rates of methane oxidation. The relative abundance of taxa associated with methanotrophy [‘Known methanotroph taxa’, verrucomicrobial methanotrophs (Verruco Mtroph), gammaproteobacterial methanotrophs (Gamma Mtroph), alphaproteobacterial methanotrophs (Alpha Mtroph)] is bifurcated from those taxa not associated with methanotrophy (‘Non-methanotroph taxa’). Error bars represent the standard error of the slope of regression obtained from observed methane oxidation within microcosms. COM, Craters of the Moon; GDS, Golden Springs; LPR, Loop Road; NGM, Ngatamariki; OKO, Orakei Korako; RTK, Rotokawa; TKA, Te Kopia; TKT, Tikitere; TOK, Tokaanu; WAM, Waimangu; WAP, Waipahihi; WHV, Whakarewarewa Village; WKT, Waikite Valley; WTV, Wairakei Thermal Valley.

Microcosm experiments illustrate that methanotrophic activity in high-temperature geothermal ecosystems correlates poorly with community abundance of known methanotrophs. Following incubation within a methane headspace, 31 of the 57 geothermal microcosms (54.3%) were identified as positive for methane oxidation (Figure 2A). Methane oxidation rates ranged from 0.5 μmol g^−1^ d^−1^ wet weight (LPR16, 70°C) to a maximum of 17.4 μmol g^−1^ d^−1^ wet weight (OKO2, 70°C; Supplementary Figure 1; Supplementary Table 1). These rates are comparable with previous studies of New Zealand geothermal areas, with maximum recorded oxidation rates of 7.0 μmol g^−1^ d^−1^ wet weight (55°C) (Sharp et al., 2012), and 20.4 μmol g^−1^ d^−1^ wet weight (37°C) or 12.7 μmol g^−1^ d^−1^ wet weight (65°C) (Sharp et al., 2014b). Other surveys of geothermal soils have reported maximum methane oxidation rates between 0.8 μmol g^−1^ d^−1^ wet weight (Italy, 50°C) (Gagliano et al., 2014) and 99 μmol g^−1^ d^−1^ wet weight (Canada, 45°C) (Sharp et al., 2014a). These rates are an order of magnitude greater than those recorded for forest soils (~0.3 nmol g^−1^ d^−1^ wet weight) (Zeng et al., 2019) and lake sediments (~300 nmol g^−1^ d^−1^ wet weight) (Bornemann et al., 2016), and comparable with methane oxidation in wetlands (17.3 μmol g^−1^ d^−1^ wet weight) (Esson et al., 2016).

Observed methane oxidation rates suggest methanotrophs are active at temperature and pH values that exceed reported values for methanotroph isolates. In this study, the fastest rates of methane oxidation were observed in microcosms incubated between 60 and 70°C (Figure 2A). Methane oxidation was also observed in six microcosms incubated at 75°C, with LPR14 being notable for the highest oxidation rate at this temperature of 3.0 μmol g^−1^ d^−1^ wet weight. To date, *Methylothermus* strain HB is reported as having the hottest growth temperature for a methanotroph (72°C), but is no longer extant (Bodrossy et al., 1999). Of extant strains, *Methylothermus thermalis* grows at 67°C (Tsubota et al., 2005), and *Methylothermus subterraneous* (Hirayama et al., 2011) and *Methylacidiphilum fumariolicum* (Pol et al., 2007) both have maximal reported growth temperatures of 65°C.

Microcosm methane oxidation across a broad pH range further suggests undescribed methanotroph activity. Methane oxidation within microcosms was observed at pH values as acidic as pH 1.5 (LPR16, 0.5 μmol g^−1^ d^−1^ wet weight) and as alkaline as pH 8.7 (WHV13, 0.5 μmol g^−1^ d^−1^ wet weight). Thermoacidophilic *Methylacidiphilum* species, previously isolated in New Zealand (Dunfield et al., 2007; Carere et al., 2017) and elsewhere (Pol et al., 2007; Islam et al., 2008), are capable of growth between pH 0.8 and pH 6.0. However, these species appear to be constrained to growth between 37 and 65°C (Houghton et al., 2019; Schmitz et al., 2021a) with the closely related acidophilic *Methylacidimicrobium* species growing to a temperature of 55°C (Picone et al., 2021b). Three acidic pH microcosms displayed substantial rates of methane oxidation at temperatures >65°C: LPR14 (75°C, pH 2.8), TKT67 (70°C, pH 2.6), and LPR16 (70°C, pH 1.5). Proteobacterial methanotrophs generally prefer neutral pH (Houghton et al., 2019), and only *Methyloferula stellata* has been shown to grow at < pH 4.0 (at pH 3.5–7.2, and 4–33°C) (Vorobev et al., 2011). The high-temperature methane-oxidizing microcosms in this study also included activity at pH 8.3 (70°C) and pH 8.7 (75°C), exceeding reported values for methanotroph isolates. *M. subterraneous*, *M. fumariolicum* and *M. thermalis* have pH maxima of 7.5, 5.8 and 7.5, respectively (Tsubota et al., 2005; Pol et al., 2007; Hirayama et al., 2011). *Methylocaldum* species are known to grow at ≤ pH 8.5 (Eshinimaev et al., 2004), but not at temperatures exceeding 62°C, while *Methylotuvimicrobium* species can grow at ≤ pH 11.0, but only to ≤45°C (Kalyuzhnaya et al., 2001). Nevertheless, methane oxidation (without growth) may be observed at temperatures exceeding those recorded for growth (Houghton, 2018), as previously demonstrated with methanogenesis (Miller et al., 1988). In these situations, growth may be limited by critical heat labile enzymes (Wiegel, 1990), or cells may use the derived energy for processes other than growth such as maintenance activities or the production of intracellular storage polymers (Linton and Cripps, 1978; Pirt, 1982).

### 3.2. Microcosm communities show similar diversity, but are enriched with known methanotrophs where high rates of methane oxidation are observed

A total of 8,605 unique OTUs were detected across all microcosms (mean of microcosm OTUs 921; s.d. = 570) (Supplementary Table 1). An assessment of community composition indicated that many of the microcosm communities were highly diverse (Supplementary Table 1; Figure 2B) and dominated by Archaea (Supplementary Table 1). A large number of OTUs across multiple microcosms were affiliated with taxa known only through DNA sequencing; in WHV18 up to 99% of OTUs were from uncultured Bacteria or Archaea, with more than 50% uncultured in 47 microcosms. This abundance of OTUs with no described or isolated representatives is typical of geothermal systems and highlights the need to characterize these systems (Inskeep et al., 2010; Rinke et al., 2013; Yasir et al., 2019).

Seven archaeal and bacterial genera were found in >90% of the geothermal microcosms: *Methylacidiphilum* (Verrucomicrobiota), *Chthonomonas* (Armatimonadota), *Thermus* (Deinococcota), *Sulfolobus* (Thermoproteota), *Thermoplasma* (Euryarchaeota), *Caldimicrobium* (Thermodesulfobacteriota), and *Alicyclobacillus* (Bacillota). However, these ubiquitous genera comprised on average < 5% of the total reads in the microcosms and, with the exception of *Methylacidiphilum*, are likely to represent cosmopolitan taxa present across NZ geothermal environments rather than contributing directly to methane consumption (Huber and Stetter, 2001; Reysenbach, 2001; da Costa et al., 2009; Albuquerque and da Costa, 2014; Kojima et al., 2016; Lee et al., 2016). No significant differences in alpha diversity were observed between ‘methane-oxidizing’ and ‘non-oxidizing’ microcosms (*p* = 0.25 for Shannon and *p* = 0.20 for Simpson). Despite the similar alpha diversity metrics, the community composition differed significantly, most notably two methanotrophic genera (*Methylacidiphilum* and *Methylothermus*), four other genera (*Thermoplasma*, *Alicyclobacillus*, *Sulfurimonas* and several species of pseudomonads) not known for a methanotrophy phenotype were significantly enriched in ‘methane-oxidizing’ communities (Table 2). We speculate that their increased abundance could be a result of positive syntrophic interactions such as increased access to C1 by-products by methylotrophic pseudomonads (Riis et al., 2003), or other metabolic by products from the heterotrophic scavengers *Thermoplasma* and *Alicyclobacillus* (Reysenbach, 2001; Karavaiko et al., 2005). Conversely, the increased abundance may be merely a co-occurrence of conducive growth conditions, e.g., the elevated atmospheric CO_2_ amended to the microcosms are likely to promote the growth of autotrophs such as *Sulfurimonas* (Han and Perner, 2015). Sulfide-oxidizing *Sulfurimonas* strains may also benefit from the production of hydrogen sulfide from methanethiol by *Methylacidiphilum* species (Lahme et al., 2020; Schmitz et al., 2022).

**Table 2:** Genera enriched in methane-oxidizing microcosms.

		Sum of normalized reads across all microcosms		
Phylum	Genus	Methane-oxidizing	Non-methane oxidizing	Value of *p*
Verrucomicrobiota	*Methylacidiphilum*	5.74%	1.63%	0.012
Euryarchaeota	*Thermoplasma*	4.25%	0.34%	<0.0001
Pseudomonadota	*Pseudomonas*	1.64%	0.21%	0.011
Pseudomonadota	*Methylothermus*	1.06%	0.05%	0.046
Pseudomonadota	*Sulfurimonas*	0.38%	0.02%	<0.0001
Bacillota	*Alicyclobacillus*	0.37%	0.24%	0.0005

The value of *p* was calculated using a Kruskal-Wallis test to determine significance of enrichment. Only taxa with an abundance of >0.1% of total normalized reads and a value of *p* of <0.05 are shown.

### 3.3. Microcosms support both aerobic and anaerobic methanotrophs

Putative aerobic and anaerobic methanotroph OTUs were detected in all geothermal microcosms, regardless of whether they oxidized methane or not, across a wide range of both temperature and pH (Supplementary Figure 3; Supplementary Table 1). All of the major clades of aerobic methanotrophs Alphaproteobacteria, Gammaproteobacteria and Verrucomicrobiales were detected across the microcosm experiment.

Within the alphaproteobacterial clade, *Methylocystis* OTUs were detected in low abundance within 19 microcosms, ranging from pH 4.2 to pH 7.7 (Supplementary Figure 3) and at *in situ* temperatures between 38.0 and 77.3°C. Currently, all cultivated *Methylocystis* strains have reported maximum growth temperatures of ≤53°C (Tsypenzhapova et al., 2007). Molecular-based surveys have detected *pmoA* sequences similar to *Methylocystis* and *Methylocapsa* in a 51°C hot spring (Zelenkina et al., 2009) and in geothermal sediments up to 76°C (Sharp et al., 2014b), while Kizilova et al. (2014) demonstrated ^14^CH_4_ bio-assimilation in hot spring samples containing Alphaproteobacteria OTUs incubated at 75°C (Kizilova et al., 2014), further supporting the possibility of thermophilic alphaproteobacterial methanotrophs within these microcosms.

**Figure 3:**
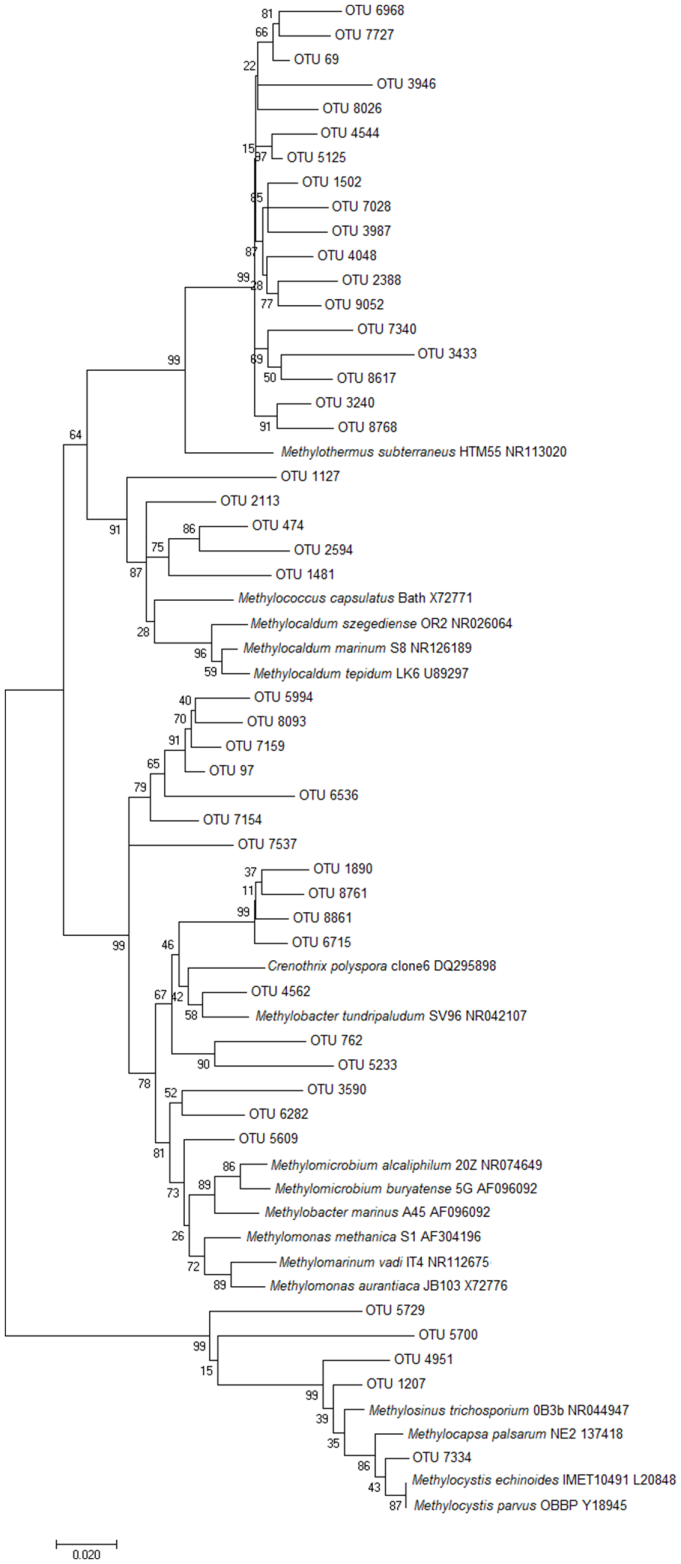
Unrooted phylogenetic tree of the putative methanotroph OTUs from Pseudomonadota. The evolutionary history was inferred using Neighbor-Joining (Saitou and Nei, 1987), and evolutionary distances were computed using the Maximum Composite Likelihood method (Tamura et al., 2004). Bootstrap values >50% are shown at the branch nodes (1000 replicates). The scale bar represents 0.02 nucleotide substitutions per site, and analyses were conducted using MEGA7 (Kumar et al., 2016).

In comparison, OTUs related to methanotrophs from Gammaproteobacteria (e.g., *Methylothermus*, *Methylococcus*, *Methylocaldum*, *Methylomonas*, *Crenothrix*, *Methylobacter*) were identified in 49 microcosms and were substantially more abundant than those from Alphaproteobacteria, with an average abundance of 0.7% of all reads, and a maximum of 11.2% of normalized reads from TOK7. These microcosms ranged from pH 1.5 to pH 9.2, and from 35.0°C to 85.0°C (Supplementary Figure 3). The majority of gammaproteobacterial methanotroph reads affiliated most closely to thermophilic *Methylothermus* strains (Tsubota et al., 2005; Hirayama et al., 2011), but the microcosms GDS1 (38.9°C, pH 7.2) and GDS2 (38.0°C, pH 7.0) also contained a small number of OTUs most similar to thermotolerant (*Methylococcus* or *Methylocaldum*) and mesophilic (*Methylomonas*, *Crenothrix* and *Methylobacter*) strains (Figure 3).

*Methylacidiphilum* strains from Verrucomicrobiota were the most ubiquitous and abundant methanotrophs detected throughout this survey; detected in every microcosm with an average abundance of 3.7% (up to a maximum of 60.7% of reads, NGM89). The majority of these OTUs were most closely related to the thermoacidophilic *M. infernorum* (Dunfield et al., 2007). Microcosms spanned temperature (35.0–85.0°C) and pH (pH 1.5–9.2) values that exceed the currently known methane-dependent growth ranges of *Methylacidiphilum* and *Methylacidimicrobium* isolates (Islam et al., 2008; Op den Camp et al., 2009; van Teeseling et al., 2014). While some of these *Methylacidiphilum* OTUs are likely relic DNA, it is also plausible that these taxa are growing/persisting at elevated temperatures on non-methane substrates, e.g., simple organic acids, alcohols, alkanes, H_2,_ H_2_S or methanethiol (Carere et al., 2017, 2021; Picone et al., 2021b; Schmitz et al., 2022, 2023) and/or oxidizing methane via a persistence strategy (Houghton, 2018).

Microcosm OKO2 was collected from a steam-influenced soil and showed the greatest rate of methane oxidation (17.4 μmol g^−1^ d^−1^ wet weight) observed in this study. The soil was moderately acidic (pH 4.3) with an *in situ* temperature of 71.6°C. Analysis of the community composition revealed very few known methanotroph OTUs. In fact, the only reads associated with a known methanotrophic phenotype were from the genus *Methylacidiphilum*, but these reads were at such low abundance (13 reads total) that they cannot be responsible for the observed rates of methane oxidation. In contrast, the OKO2 microcosm was dominated (65.4% of total normalized reads) by Bathyarchaeota sequences. Recently published genomes of this phylum suggest that some strains may be capable of anaerobic methanotrophy (Evans et al., 2015). Despite this microcosm being incubated aerobically, it is plausible these candidate taxa may be responsible for the observed rates of methane oxidation. Other known anaerobic methanotrophs, *Methanoperedens* and ANME-1, were also detected along with several known aerobic methanotrophs at low abundance within a single microcosm (GDS2).

A minority of microcosms displayed rapid rates of methane oxidation despite a low abundance of known/putative methanotrophs (Figure 2B). TKT67 oxidized 9.4 μmole methane g^−1^ d^−1^ wet weight (the second fastest rate measured) and TKT68 oxidized 7.1 μmole methane g^−1^ d^−1^ wet weight. Both microcosms were dominated by Archaea (Thermoproteota and Nitrososphaerota, respectively) and both had ~0.6% of sequences classified to *Methylacidiphilum* and ~ 0.1% to *Methylothermus* (Figure 2B; Supplementary Table 2). In addition, TOK17 oxidized 5.5 μmole methane g^−1^ d^−1^ wet weight, but only 4.1% of sequences were classified as *Methylothermus* and 0.2% to *Methylacidiphilum* (Supplementary Table 1). Although community abundance does not necessarily correlate to methanotroph activity (Wigley et al., 2022), collectively these data suggest hitherto undescribed methanotrophs may be responsible for some of the methane-oxidizing activity observed.

### 3.4. Metatranscriptomic analysis of enrichments reveals active community members

To investigate the ability for thermophilic microcosms to propagate within growth media, a series of enrichment experiments were performed. Thirty-one microcosms that oxidized methane (>0.5 μmole g^−1^ d^−1^) were inoculated into different growth media for methanotroph enrichment experiments (*n* = 71 enrichments, Supplementary Figure 4; Supplementary Table 2). Ten microcosms which displayed methane oxidation (>0.5 μmole g^−1^ d^−1^) were unable to support this activity as enrichments (Figure 2; Supplementary Table 4). A similar loss of activity at elevated temperatures was seen in enrichment microcosms from landfill cover soil (Reddy et al., 2019). In this study, at 50°C methylotrophic communities shifted from primarily *Methylocaldum* species to a combination of *Methylobacter*, *Methylocystis* and non-methane-oxidizing Methylophilaceae, although these were presumably dormant organisms (Reddy et al., 2019). The loss of methane oxidation capacity is likely due to poor mass-transfer of methane (e.g., diffusivity, solubility) within a liquid medium at high temperatures (Castro et al., 1995; Duan and Mao, 2006), or an inability to provide conducive growth conditions in the growth medium, e.g., rare earth elements (lanthanides) (Keltjens et al., 2014). The lanthanides are essential as a cofactor in XoxF methanol dehydrogenases, which are commonly present in the genomes of both methanotrophic and methylotrophic bacteria, often in addition to MxaF methanol dehydrogenases (Chistoserdova, 2016). XoxF is essential for growth in verrucomicrobial *Methylacidiphilum* (Pol et al., 2013) and *Methylacidimicrobium* strains (Schmitz et al., 2021b), but only a few proteobacterial *Methylosinus* (Kato et al., 2020) and *Methylocella* (Crombie, 2022) strains. In this study, lanthanides were only added to V4 medium for enrichment of *Methylacidiphilum* strains (Supplementary Material 1).

To investigate the activity of the methane-oxidizing microbial communities, two enrichments (GDS1 and TOK7) were selected for metatranscriptomic analysis on the basis of observed methane-oxidizing activity, the prevalence of methanotroph taxa within initial microcosms, and continued methane oxidation in the enrichments. Sample GDS1 was collected from a site at Golden Springs (pH 7.2, 38.9°C), with the microcosm initially incubated at 50°C to promote any thermophilic methanotrophic activity. The GDS1 enrichment oxidized methane at a rate of 24.5 μmoles d^−1^ in NMS medium (Supplementary Table 4). The 16S rRNA gene sequences were dominated by Pseudomonadota (78.3% of total reads), with 5.5% of reads classified to *Methylococcaceae* (Supplementary Table 3). A total of 30.0 Gb trimmed and quality checked reads from three biological replicates of GDS1 (10.0 ± 0.7 Gb/sample) were obtained. More details are in Supplementary Table 5.

**Figure 4:**
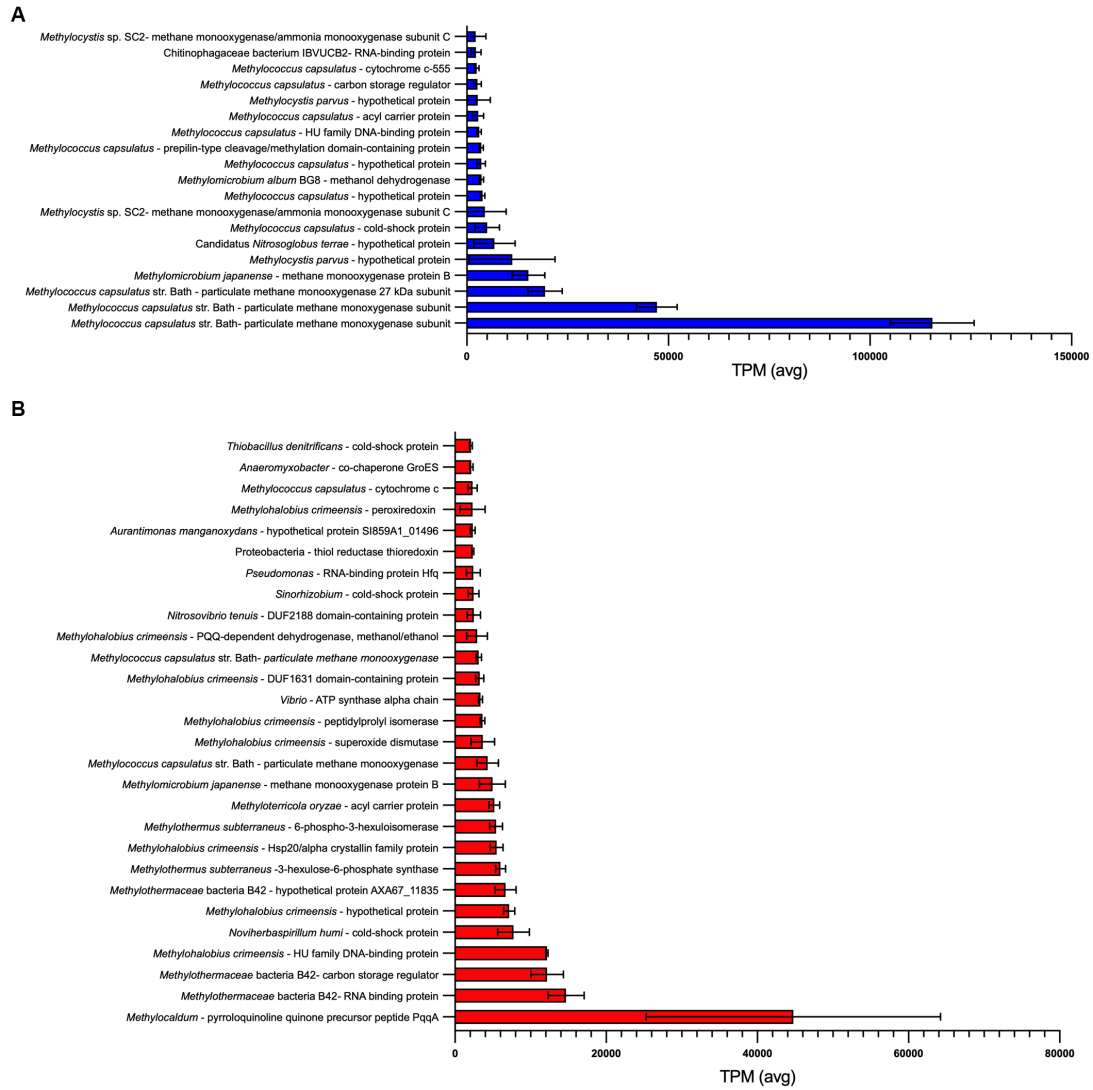
Most abundant transcripts identified within **(A)** GDS1 and **(B)** TOK7 enrichments. Transcripts with an average transcripts per million (TPM) > 2000 across three biological replicates are shown, error bars represent 1 standard deviation. Identity is based on a BLASTx search using the NCBI non-redundant (nr) database (Supplementary Tables 5, 6).

For GDS1, 18 of the 20 most abundant transcripts were assigned to *Methylococcus*, *Methylomicrobium* or *Methylocystis* species (Figure 4A; Supplementary Table 5). The remaining transcripts predominantly encoded for proteins assigned to Alphaproteobacteria (*Rhodopseudomonas*, 19.6%; *Aurantimonas*, 9.7%; *Novosphingobium*, 7.5%), and Actinomycetota (*Streptomyces*, 6.4%). Interestingly, these transcripts were not representative of the initial 16S rRNA gene sequences obtained from the original microcosm, which were predominately *Pseudomonas* spp. (46.9% of total normalized reads), and with no OTU reads affiliated to *Rhodopseudomonas*, *Aurantimonas* or *Novosphingobium*. This may be due to dormancy as up to 80% of soil microorganisms identified through DNA sequencing are thought to be in a low state of metabolic activity at any given time (Lennon and Jones, 2011).

Particulate methane monooxygenase proteins comprised between 18.5 to 22.9% of all transcripts in GDS1 replicates, and multiple methanol dehydrogenase proteins comprised 4.7 to 8.4% of all transcripts. On the basis of observed transcripts, enrichments may also be capable of methylamine oxidation to formaldehyde (K15229), as observed in multiple methanotrophic strains, including *Methylomonas*, *Methylococcus* and *Methylocella* species (Bowman et al., 1993; Dedysh et al., 2005) and methylotrophic Methylophilaceae spp. (Chistoserdova, 2011). The genomes of several *Methylacidiphilum* strains also contain methylamine dehydrogenase genes (Dunfield et al., 2007; Hou et al., 2008; Op den Camp et al., 2009). However, while trimethylamine could be oxidized by trimethylamine dehydrogenase (K00317), no transcripts encoding enzymes capable of oxidizing the resulting dimethylamine were detected. Transcripts for multiple potential mechanisms for formaldehyde oxidation were expressed, including glutathione-dependent formaldehyde dehydrogenases, and tetrahydrofolate- and tetrahydromethanopterin-linked pathways (Chistoserdova, 2011). However, transcripts for a membrane-associated quinoprotein formaldehyde dehydrogenase, previously reported to be the major formaldehyde-oxidizing enzyme in *Methylococcus capsulatus* during pMMO expression (Zahn et al., 2001), were not detected in this study. All genes encoding enzymes required for the RuMP and CBB cycles for carbon assimilation were identified, but a complete serine cycle pathway was not detected. Two essential modules were not detected in transcripts; K00830, which represents both serine to hydroxypyruvate and glycoxylate to pyruvate; and K08692, which represents malate-CoA ligase.

Consistent with other methane-oxidizing communities at the oxic/anoxic interface (Wigley et al., 2022), GDS1 enrichments were putatively capable of assimilatory and (partial) dissimilatory nitrate reduction (NMS medium contains 0.6 g/L^−1^ NO_3_). Multiple pathways were identified in GDS1 transcriptomes for both dissimilatory and assimilatory nitrate reduction to ammonia using genes encoding the NarGHJI (K00370), NapAB (K02567) and NasAB (K00372) forms of nitrate reductases, although these were not identified as from methanotrophs. Nitrite could be converted to ammonia by various forms of nitrite reductase, using either NADH (NirBD, K00362), cytochrome-c552 (NrfAH, K03385), NAD(P)H (NIT-6, K17877) or ferredoxin (NirA, K00366) as co-factors. However, genes encoding NO-forming nitrite reductase (NirK, K00368, or NirS, K15864) were not detected. One possible explanation could be that methanotrophy is mediated via a similar nitrate-dependent mechanism to the mesophilic genera *Methylomirabilis* (phylum NC10) (Guerrero-Cruz et al., 2019) via yet-to-be-described taxa when oxygen at the oxic-anoxic interface becomes limited. GDS1 also displayed evidence for nitrogen reduction to ammonia via the activity of a molybdenum-iron nitrogenase (NifDKH, K02586).

Sample TOK7 was collected from a clay soil site at Tokaanu (pH 6.8, 63.7°C, Supplementary Table 1). The TOK7 enrichment suspended in ANMS medium oxidized CH_4_ at a rate of 3.5 μmoles d^−1^ (Supplementary Table 4) while incubated at 60°C. The archaeal phylum Bathyarchaeota, which potentially includes anaerobic methanotrophs (Evans et al., 2015), was the most abundant phylum detected via 16S rRNA gene sequences (27.9%). Also noteworthy was an abundance (11.2%) of sequences classified into the gammaproteobacterial family of thermophilic methanotrophs Methylothermaceae (Houghton et al., 2019; Supplementary Table 3).

A total of 31.7 Gb trimmed and quality checked reads from three biological replicates of TOK7 (10.6 ± 1.3 Gb/sample) were obtained. More details are in Supplementary Table 6. The most highly expressed genes were related to known methanotroph taxa (Figure 4B). In TOK7 samples, 13 of the 28 most abundant transcripts were assigned to strains from Methylothermaceae, and another six to Methylococcaceae (Supplementary Table 6). However, there were only 109 transcripts in total from methanotrophs (0.2%), including 16 from *Methylocystis* or *Methylosinus*. No transcripts from Bathyarchaeota were identified. The remaining transcripts included genes encoding for proteins classified as *Pseudomonas* (Gammaproteobacteria, 7.8%), *Anaeromyxobacter* (Deltaproteobacteria, 5.2%), *Streptomyces* (Actinomycetota, 5.2%), *Mycobacterium* (Actinomycetota, 5.0%), and *Rhodopseudomonas* (Alphaproteobacteria, 5.0%). Similar to GDS1 samples, this did not reflect the original community 16S rRNA gene sequences, which contained only six *Pseudomona*s OTUs from 149,400 normalized reads, and none of the other aforementioned genera. Although transcriptome analysis specifically identifies ‘active’ members in a mixed community, taxonomic assignments based on community protein transcripts should also be assessed with caution, as there is known bias within the NCBI database for Pseudomonadota, Actinomycetota, Bacteroidota, and Bacillota (Rinke et al., 2013). Furthermore, high conservation of protein domains across taxa (or variability within families) may further complicate taxonomic placement of transcripts (Mount, 2004).

Consistent with the rapid rates of methane oxidation observed within TOK7 enrichments (Supplementary Figure 5), genes involved in one-carbon metabolism were highly expressed. The most highly expressed gene (5.8% of transcripts) encoded a methylotrophy-associated pyrroloquinoline quinone (PQQ) precursor peptide, PqqA (Chistoserdova et al., 2003). PQQ-dependent dehydrogenases (methanol/ethanol family, *Methylohalobius*) were also abundant (0.4–0.8% of transcripts) (Supplementary Table 6). Genes encoding the particulate form of methane monooxygenase (*Methylomicrobium*/*Methylococcus*/*Methylocystis*) comprised between 0.9 and 1.8% of transcripts (Supplementary Table 6). These did not match with the methanotrophs identified during 16S rRNA sequencing (primarily *Methylothermus,* with nine *Methylocystis* reads). Formaldehyde could be assimilated and detoxified via S-(hydroxymethyl) glutathione synthase (K03396), a formaldehyde activating enzyme (K10713) for the tetrahydromethanopterin pathway, or by 3-hexulose-6-phosphate synthase (K08093) for the RuMP pathway. Carbon assimilation within the TOK7 community may also be achieved via the CBB cycle, as all genes for this pathway were identified from the assembly. In contrast, the serine cycle for carbon assimilation was not complete, as genes encoding malate-CoA ligase (K08692), malyl-CoA lyase (K08691) and an alanine/serine transaminase (K00830) were all absent from the transcripts. No genes encoding enzymes for the oxidation of mono-, di- or trimethylamine were found in the TOK7 transcripts, and the only evidence for methanogenesis were genes encoding two subunits (of six) of heterodisulfide reductase.

TOK7 enrichments were putatively capable of assimilatory and (partial) dissimilatory nitrate reduction, although these were not associated with methanotrophs (ANMS medium contains 0.15 g/L^−1^ NO_3_). The TOK7 community expressed *narGHI* genes for dissimilatory nitrate reduction to nitrite and could potentially convert this nitrite to ammonia via NirBD (K00362) or NfrAH (K03385) nitrite reductases. The community was also capable of assimilatory nitrate reduction using NAD(P)H-dependent nitrate reductase (K10534) or the NasAB (K00372) nitrate reductase, and finally converting the resultant nitrite to ammonia using nitrite reductases NIT-6 (K17877) or NirA (K00366), which use NAD(P)H or ferredoxin, as co-factors, respectively (Exley et al., 1993; Takahashi et al., 2001). No genes for the fixation of nitrogen via nitrogenases *nifDKH* or *anfHDGK* were identified in TOK7.

## 4. Conclusion

The data presented in this study broadly supports the premise that thermophilic CH_4_ oxidation is widespread in New Zealand geothermal fields, with more than 50% of the methanotroph-targeting microcosms exhibiting methane oxidation at temperatures greater than 38°C and as hot as 75°C. Methane oxidation rates up to 17.4 μmol g^−1^ d^−1^ wet weight indicate that this is an important sink for CH_4_ emitted from geological sources. Microbial communities within microcosms were highly diverse, with few ubiquitous genera, but both known aerobic methanotrophs and putative anaerobic methanotrophs were identified via 16S rRNA gene sequencing of microcosms. To investigate this further, we attempted to enrich methanotrophic consortia from 32 thermophilic methane-oxidizing microcosms (< 75°C) but were unable to maintain many of the cultures. For enrichments that did oxidize methane, observed rates correlated poorly to the original microcosm, or to the presence of known methanotroph OTUs (as predicted via 16S rRNA gene amplicon sequencing). The cessation of CH_4_ oxidation in these enrichments was possibly due to the poor solubility of CH_4_/O_2_ at high temperatures. Consequently, poor mass transfer of these gases into the growth medium may be hampering attempts to cultivate and isolate novel thermophilic methane-oxidizing bacteria using traditional batch cultivation techniques. While we present evidence for the widespread occurrence of methane oxidation in New Zealand geothermal environments, there is also evidence of the presence of methanotrophs in high temperature environments globally (Houghton et al., 2019; Wang et al., 2022; Zhou et al., 2022).

Finally, metatranscriptomic analysis of enrichments actively oxidizing methane showed genes involved in carbon metabolism were highly transcribed. At mesophilic temperatures (GDS1, 37°C), these genes were most similar to *Methylococcaceae* species, while at hotter temperatures (TOK7, 46°C), the majority of transcripts were associated with the moderately thermophilic *Methylothermaceae*. The important role of methanotrophs in nitrogen metabolism under oxic conditions (Hoefman et al., 2014) was also highlighted; both GDS1 and TOK7 communities were carrying out assimilatory and dissimilatory nitrate reduction, although a denitrification pathway was not complete in either set of transcripts. Collectively, this study expands knowledge of thermophilic methanotrophy in geothermal areas and suggests that hitherto unidentified methanotrophs may be responsible for some of this activity. A closer examination of the mechanisms involved in control of expression of methane and nitrogen metabolism pathways will augment our understanding of how methanotrophs survive and flourish in these ecosystems.

## Data availability statement

The datasets presented in this study can be found in online repositories. The names of the repository/repositories and accession number(s) can be found at: https://www.ncbi.nlm.nih.gov/, PRJNA766707, https://www.ncbi.nlm.nih.gov/, PRJNA546003, https://doi.org/10.5281/zenodo.5535083, 5535083.

## Author contributions

KH: Conceptualization, Formal analysis, Funding acquisition, Investigation, Methodology, Visualization, Writing – original draft, Writing – review & editing. CC: Conceptualization, Funding acquisition, Methodology, Visualization, Writing – review & editing. MS: Conceptualization, Funding acquisition, Methodology, Supervision, Visualization, Writing – review & editing. IM: Conceptualization, Methodology, Supervision, Writing – review & editing.

## Funding

This work was supported by the GNS Science New Zealand’s Geothermal Future Research Programme, the Royal Society of New Zealand (Marsden Grant GNS1601 to CC) and a Freemasons New Zealand Postgraduate Scholarship to KH.

## Conflict of interest

The authors declare that the research was conducted in the absence of any commercial or financial relationships that could be construed as a potential conflict of interest.

## Publisher’s note

All claims expressed in this article are solely those of the authors and do not necessarily represent those of their affiliated organizations, or those of the publisher, the editors and the reviewers. Any product that may be evaluated in this article, or claim that may be made by its manufacturer, is not guaranteed or endorsed by the publisher.
