# Supplementary material for: Thermophilic methane oxidation is widespread in Aotearoa-New Zealand geothermal fields
Source: Front Microbiol. 2023 Aug 31;14:1253773. doi: 10.3389/fmicb.2023.1253773 (PMC10502179; doi:10.3389/fmicb.2023.1253773)
Supplement: Supplementary file 1 [file Data_Sheet_1.docx]

Supplementary Material

Supplementary Table 1. Physical characteristics, methane oxidation, and key DNA sequencing results of microcosms from geothermal sites.

Gas measurements marked * are taken from the 1000 Springs dataset (Power et al., 2018) at the same location. The methane oxidation value for the Waipahihi sample WAP11 is from an enrichment culture, and is in μmol/day.

| **Geothermal field** | **Location (lat, long)** | **Microcosm ID** | **Temperature (°C)** | **pH** | **ppm CH_4_ *in situ*** | **Sample description** | **Incubation temperature (°C)** | **Methane oxidation rate**  **(µmol g^-1^ d^-1^)** | **Number of OTUs** | **Most abundant phylum** | **Percentage of *Alpha-proteobacteria* methanotrophs** | **Percentage of *Gamma-proteobacteria* methanotrophs** | **Percentage of *Verrucomicrobiota* methanotrophs** |
| --- | --- | --- | --- | --- | --- | --- | --- | --- | --- | --- | --- | --- | --- |
| Craters of the Moon | 38°38'41"S 176°04'18"E | COM19 | 70.2 | 7.6 |  | Orange-red clay soil | 70 | 0.00 | 731 | *Bathyarchaeota* | 0.009 | 0.003 | 0.126 |
|  |  | COM20 | 76.0 | 6.4 |  | Steaming pink clay | 70 | 0.00 | 1130 | *Bathyarchaeota* | 0 | 0.003 | 0.173 |
| Golden Springs | 38°38'41"S 176°04'18"E | GDS1 | 38.9 | 7.2 |  | Water | 50 | 1.35 | 1527 | *Pseudomonadota* | 0 | 5.483 | 8.689 |
|  |  | GDS2 | 38.0 | 7.0 |  | Sediment | 50 | 1.89 | 3140 | *Pseudomonadota* | 0.002 | 1.465 | 0.034 |
| Loop Road | 38°24'50"S 176°21'41"E | LPR14 | 85.0 | 2.8 |  | Thick black clay | 75 | 2.96 | 547 | *Thermoproteota* | 0 | 0.146 | 2.657 |
|  |  | LPR16 | 67.4 | 1.5 | 1593.1 | Crumbly brown loam | 70 | 0.57 | 1010 | *Bathyarchaeota* | 0.001 | 0.058 | 1.609 |
|  |  | LPR17 | 59.4 | 1.6 | 3896.2 | Loam and clay | 60 | 1.00 | 1715 | *Nitrososphaerota* | 0 | 0.318 | 0.393 |
|  |  | LPR19 | 50.0 | 5.4 | 9.2 | Crumbly loam | 50 | 0.00 | 933 | *Nitrososphaerota* | 0.024 | 0.001 | 0.290 |
| Ngatamariki | 38°31'00"S 176°10'36"E | NGM89 | 70.0 | 3.0 |  | Beige to orange soil | 60 | 1.90 | 391 | *Verrucomicrobiota* | 0 | 0.015 | 60.714 |
|  |  | NGM90 | 51.0 | 7.0 |  | White surface biomass | 60 | 0.00 | 1096 | *Thermotogota* | 0.001 | 0.013 | 0.004 |
|  |  | NGM91 | 60.0 | 7.0 |  | Sediment | 60 | 1.63 | 451 | *Armatimonadota* | 0 | 5.148 | 0.017 |
| Orakei Korako | 38°28'26"S 176°08'43"E | OKO2 | 71.6 | 4.3 | 5.9 | Steaming clay/sand | 70 | 17.36 | 288 | *Bathyarchaeota* | 0 | 0 | 0.009 |
| Rotokawa | 38°37'37"S 176°11'45"E | RTK23 | 77.3 | 2.1 |  | Loam + brown sand | 75 | 0.00 | 1481 | *Thermoproteota* | 0.006 | 0.058 | 0.677 |
| Te Kopia | 38°24'26"S 176°12'32"E | TKA8 | 75.3 | 4.7 | 3.4 | Sinter + orange clay | 75 | 0.58 | 1099 | *Chloroflexota* | 0.001 | 0.003 | 0.028 |
|  |  | TKA9 | 66.3 | 4.2 | 25.2 | Red clay/sandy loam | 70 | 2.50 | 222 | *Bathyarchaeota* | 0.003 | 0.001 | 0.037 |
|  |  | TKA10 | 68.1 | 4.2 | 98.6 | Purple clay | 70 | 0.00 | 1057 | *Nitrososphaerota* | 0.002 | 0.037 | 0.501 |
|  |  | TKA11 | 51.1 | 4.5 |  | Green orange clay | 50 | 0.00 | 455 | *Nitrososphaerota* | 0 | 0 | 0.003 |
|  |  | TKA12 | 85.3 | 4.4 |  | Orange clay | 75 | 0.00 | 260 | *Thermoproteota* | 0 | 0 | 0.009 |
|  |  | TKA13 | 65.6 | 4.1 |  | Pink clay | 70 | 0.77 | 299 | *Bathyarchaeota* | 0 | 0.001 | 0.015 |
|  |  | TKA14 | 77.4 | 4.6 | 4.6 | Orange crumbly clay | 75 | 0.00 | 716 | *Nitrososphaerota* | 0 | 0.005 | 0.077 |
|  |  | TKA15 | 70.3 | 5.1 | 40.5 | Orange to white clay | 70 | 0.67 | 290 | *Armatimonadota* | 0 | 0.002 | 0.021 |
|  |  | TKA16 | 71.6 | 5.0 | 14.9 | Brown loam | 70 | 1.40 | 284 | *Bathyarchaeota* | 0.001 | 0 | 0.005 |
|  |  | TKA17 | 60.7 | 5.3 | 32.7 | Dark brown soil | 60 | 0.00 | 931 | *Nitrososphaerota* | 0.052 | 0.027 | 8.598 |
| Tikitere | 38°03'54"S 176°21'38"E | TKT67 | 75.8 | 2.6 | 13.8 | Liquid mud | 70 | 9.35 | 1816 | *Thermoproteota* | 0 | 0.111 | 0.641 |
|  |  | TKT68 | 35.0 | 3.0 |  | Crusted mud | 50 | 7.05 | 1759 | *Nitrososphaerota* | 0.009 | 0.098 | 0.683 |
| Tokaanu |  | TOK5 | 82.5 | 7.9 | 10.1 | Clay/loam | 80 | 0.00 | 1647 | *Nitrososphaerota* | 0 | 0.655 | 0.714 |
|  | 38°58'01"S 175°45'54"E | TOK6 | 74.6 | 7.6 |  | Clay/loam | 70 | 0.00 | 1379 | *Pseudomonadota* | 0 | 0.023 | 1.448 |
|  |  | TOK7 | 63.7 | 6.8 |  | Clay | 60 | 4.40 | 1029 | *Bathyarchaeota* | 0.006 | 11.225 | 17.946 |
|  |  | TOK8 | 69.0 | 7.0 |  | Brown clay | 70 | 0.00 | 1096 | *Pseudomonadota* | 0.006 | 0.025 | 0.831 |
|  |  | TOK9 | 64.0 | 6.7 |  | Dry sandy soil | 60 | 0.00 | 1413 | *Nitrososphaerota* | 0.003 | 0.005 | 0.289 |
|  |  | TOK10 | 58.3 | 6.9 |  | Sediment | 60 | 2.72 | 1461 | *Nitrososphaerota* | 0 | 0.402 | 0.081 |
|  |  | TOK11 | 63.0 | 9.2 |  | Very fine silky clay | 60 | 0.00 | 1744 | *Nitrososphaerota* | 0 | 0.094 | 0.629 |
|  |  | TOK12 | 62.5 | 8.1 |  | Sediment | 60 | 1.51 | 681 | *Parcubacteria* | 0 | 10.234 | 1.857 |
|  |  | TOK13 | 82.3 | 7.9 | 4.8 | Clay/loam | 80 | 0.00 | 1305 | *Thermoproteota* | 0 | 0.027 | 0.049 |
|  |  | TOK14 | 71.7 | 5.4 |  | Red/brown clay | 70 | 0.00 | 1805 | *Nitrososphaerota* | 0.001 | 0.079 | 0.720 |
|  |  | TOK15 | 69.6 | 6.2 |  | Steam affected clay | 70 | 0.58 | 1003 | *Nitrososphaerota* | 0 | 0.003 | 0.033 |
|  |  | TOK16 | 76.4 | 6.1 | 21.3 | Pink/brown/yellow clay | 80 | 0.00 | 1209 | *Nitrososphaerota* | 0 | 0.456 | 6.424 |
|  |  | TOK17 | 63.5 | 7.7 |  | Pink gritty sediment | 60 | 5.48 | 594 | *Armatimonadota* | 0 | 4.104 | 0.258 |
| Waimangu | 38°17'07"S 176°23'13”E | WAM36 | 73.0 | 4.6 |  | Pink clay | 75 | 1.67 | 1195 | *Thermoproteota* | 0 | 0.019 | 1.147 |
| Waipahihi | 38°42’30"S 176°05'08"E | WAP11 | 45.0 | 6.7 |  | Orange sediment | 46 | **4.55** | 1558 | *Deinococcota* | 0.006 | 0.012 | 0.524 |
| Whakarewarewa Village | 38°09'41"S 176°15'24"E | WHV8 | 80.6 | 7.0 | 112* | Grey sediment | 68 | 0.00 | 442 | *Aquificota* | 0 | 0 | 0.005 |
|  |  | WHV12 | 58.5 | 3.0 | 2.4 | Steaming sand/loam | 60 | 1.86 | 248 | *Verrucomicrobiota* | 0 | 0.001 | 50.602 |
|  |  | WHV13 | 84.8 | 8.7 | 12.3* | Grey sediment | 75 | 0.48 | 537 | *Aquificota* | 0 | 0.001 | 0.416 |
|  |  | WHV14 | 75.1 | 8.3 | 7.2* | Soft grey sediment | 75 | 0.00 | 323 | *Thermodesulfobacteriota* | 0 | 0 | 0.007 |
|  |  | WHV15 | 82.1 | 6.3 | 20.2* | Brownish grey biomass | 75 | 0.68 | 433 | *Thermoproteota* | 0.001 | 0.004 | 0.110 |
|  |  | WHV16 | 62.5 | 5.6 |  | Sediment | 60 | 1.53 | 730 | *Chlorobiota* | 0 | 0.001 | 0.002 |
|  |  | WHV18 | 55.7 | 2.5 |  | White and brown soil | 60 | 1.84 | 276 | *Euryarchaeota* | 0 | 0.003 | 0.496 |
|  |  | WHV20 | 64.5 | 5.3 |  | Sediment | 70 | 0.00 | 316 | *Aquificota* | 0 | 0.001 | 0.006 |
| Waikite Valley | 38°19'17"S 176°18'40"E | WKT43 | 72.4 | 7.8 | 47.2 | Reddish brown clay | 70 | 0.00 | 720 | *Nitrososphaerota* | 0.001 | 0.002 | 0.007 |
|  |  | WKT44 | 60.0 | 8.3 | 2.8 | Red moist clay | 60 | 0.00 | 570 | *Deinococcota* | 0 | 0.003 | 0.003 |
|  |  | WKT45 | 71.1 | 8.3 | 2.3 | Red clay | 70 | 0.94 | 452 | *Aigarchaeota* | 0 | 0.001 | 0.013 |
|  |  | WKT46 | 39.7 | 5.8 | 56.8 | Brown sandy soil | 40 | 0.00 | 648 | *Chloroflexota* | 0.001 | 0 | 6.030 |
|  |  | WKT47 | 68.4 | 6.5 | 732.5 | Sandy soil/red clay | 70 | 0.00 | 585 | *Nitrososphaerota* | 0.114 | 0 | 13.936 |
| Wairakei  Thermal Valley | 38°37'24"S 176°05'32"E | WTV1 | 69.5 | 4.0 | 3.3 | Pink clay | 70 | 5.34 | 1138 | *Bathyarchaeota* | 0 | 0.006 | 12.021 |
|  |  | WTV2 | 73.2 | 7.7 | 3 | Black loam/pink clay | 75 | 1.40 | 1135 | *Bathyarchaeota* | 0.011 | 0.013 | 10.381 |
|  |  | WTV3 | 79.5 | 3.7 | 1.8 | White soil/pink clay | 75 | 0.00 | 818 | *Bathyarchaeota* | 0 | 0.010 | 0.576 |
|  |  | WTV4 | 65.3 | 4.0 |  | Reddish clay | 70 | 1.73 | 402 | *Bathyarchaeota* | 0 | 0 | 0.639 |


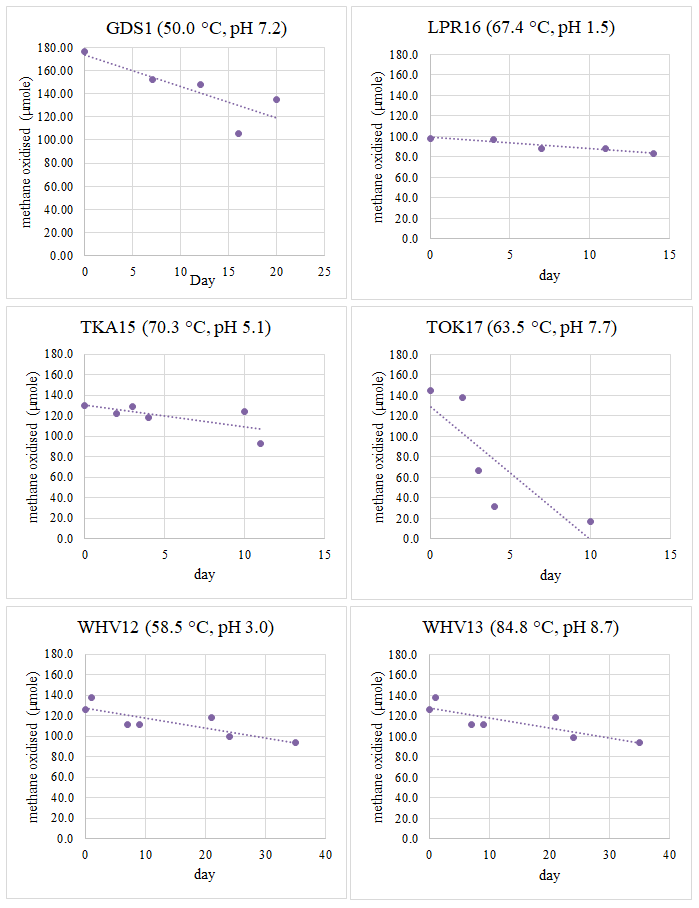


Supplementary Figure 1. Methane oxidation rates for selected soil microcosms.


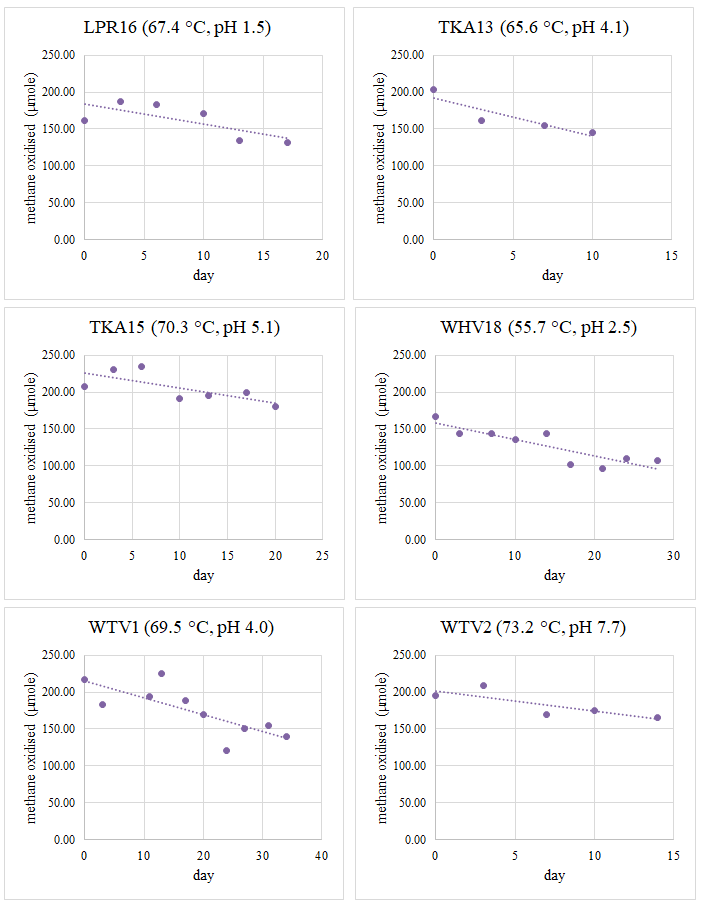


**Supplementary Figure 2. Methane oxidation rates for selected enrichments.**


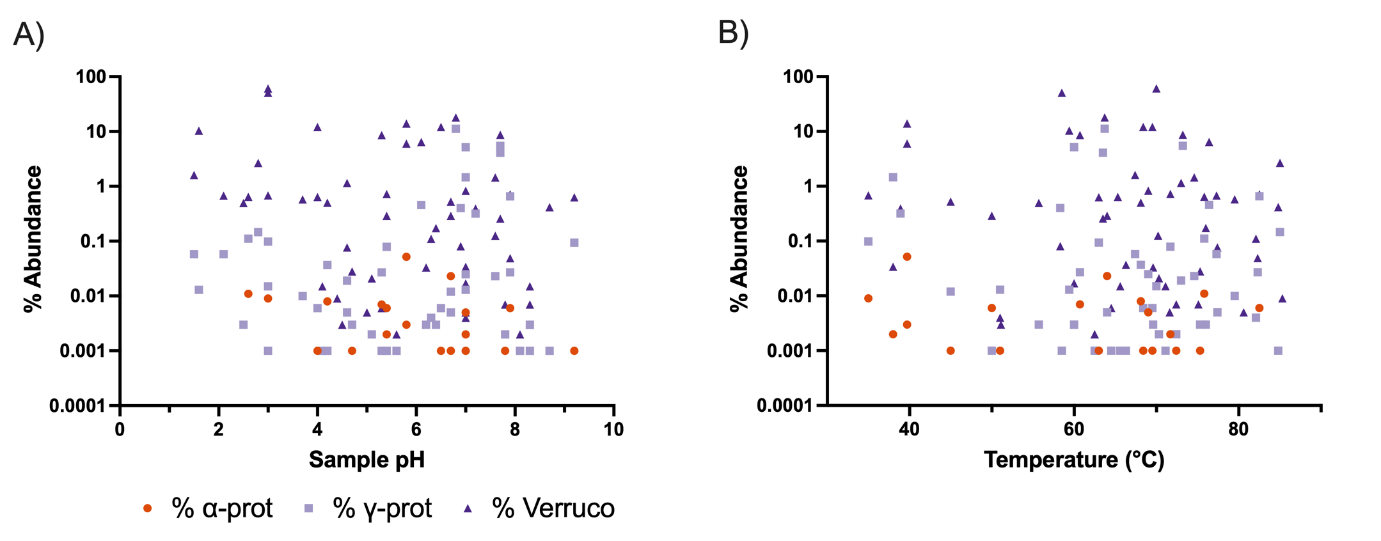


Supplementary Figure 3. Scatter plots of pH (A) and temperature (B) versus proportion of identified aerobic methanotrophs from total normalised 16S rRNA gene sequences.

Symbol shape and colour indicates phylogenetic affiliation of methanotrophs: Alphaproteobacteria, orange circles; Gammaproteobacteria, light purple squares; Verrucomicrobiota, dark purple triangles. Temperatures plotted reflect the in situ field site sample temperatures.

**Supplementary Table 2. Geothermal microcosms selected for methanotroph enrichment.**

Initial sample pH and putative methanotrophic genera identified via microbial community analysis are listed along with cultivation media and pH chosen for methanotrophic enrichments. *, samples of neutral or alkaline pH where more than 0.01 % of 16S rRNA gene sequences were identified as *Methylacidiphilum*, but samples were not inoculated into V4 medium.

| **Sample location** | **Microcosm ID** | **Microcosm pH** | **Methanotrophic genera identified**  **(> 0.01 % of total 16S rRNA**  **gene sequences)** | **Culture media** | **pH of media** | **Incubation temperature (°C)** |
| --- | --- | --- | --- | --- | --- | --- |
| Golden Springs | GDS1 | 7.2 | *Methylococcus, Methylomonas,*  *Methylacidiphilum* | mNMS, NMS, dNMS | 7.0 | 50 |
| Golden Springs | GDS2 | 7.0 | *Methylobacter, Methylococcus, Methylomonas, Methylacidiphilum* | mNMS, NMS, dNMS | 7.0 | 50 |
| Loop Road | LPR14 | 2.8 | *Methylothermus, Methylacidiphilum* | mNMS, V4 | 3.0 | 75 |
| Loop Road | LPR16 | 1.5 | *Methylothermus, Methylacidiphilum* | mNMS, V4 | 1.5 | 70 |
| Loop Road | LPR17 | 1.6 | *Methylothermus, Methylacidiphilum* | mNMS, V4 | 1.5 | 60 |
| Ngatamariki | NGM89 | 3.0 | *Methylothermus, Methylacidiphilum* | mNMS, V4 | 3.0 | 60 |
| Ngatamariki | NGM91 | 7.0 | *Methylothermus, Methylacidiphilum* | mNMS, mmj, ANMS | 7.0 | 60 |
| Orakei Korako | OKO2 | 4.3 | None | mNMS, V4 | 4.5 | 70 |
| Te Kopia | TKA8 | 4.7 | *Methylacidiphilum* | mNMS, V4 | 5.0 | 75 |
| Te Kopia | TKA9 | 4.2 | *Methylacidiphilum* | mNMS, V4 | 4.2 | 70 |
| Te Kopia | TKA13 | 4.1 | *Methylacidiphilum* | mNMS, V4 | 4.0 | 70 |
| Te Kopia | TKA15 | 5.1 | *Methylacidiphilum* | mNMS, V4 | 5.0 | 70 |
| Te Kopia | TKA16 | 5.0 | None | mNMS, V4 | 5.0 | 70 |
| Tikitere | TKT67 | 2.6 | *Methylocystis, Methylothermus, Methylacidiphilum* | mNMS, V4 | 2.5 | 75 |
| Tikitere | TKT68 | 3.0 | *Methylothermus, Methylacidiphilum* | mNMS, V4 | 3.0 | 37 |
| Tokaanu | TOK7 | 6.8 | *Methylothermus, Methylacidiphilum* | mNMS, mmj, ANMS | 7.0 | 60 |
| Tokaanu | TOK10 | 6.9 | *Methylothermus, Methylacidiphilum* | mNMS, mmj, ANMS | 7.0 | 60 |
| Tokaanu | TOK12 | 8.1 | *Methylothermus, Methylacidiphilum* | mNMS, mmj, ANMS | 8.0 | 60 |
| Tokaanu | TOK15* | 6.2 | *Methylacidiphilum* | mNMS, ANMS | 6.0 | 70 |
| Tokaanu | TOK17 | 7.7 | *Methylothermus, Methylacidiphilum* | mNMS, mmj, ANMS | 7.5 | 60 |
| Waimangu | WAM36 | 4.6 | *Methylothermus, Methylacidiphilum* | mNMS, V4 | 4.5 | 70 |
| Waipahihi | WAP11* | 6.7 | *Methylacidiphilum* | mNMS, ANMS | 7.0 | 46 |
| Whakarewarewa Village | WHV12 | 3 | *Methylacidiphilum* | mNMS, V4 | 3.0 | 60 |
| Whakarewarewa Village | WHV13* | 8.7 | *Methylacidiphilum* | mNMS, ANMS | 8.5 | 75 |
| Whakarewarewa Village | WHV15* | 6.3 | *Methylacidiphilum* | mNMS, ANMS | 6.0 | 75 |
| Whakarewarewa Village | WHV16* | 5.6 | *Methylacidiphilum* | mNMS, ANMS | 5.5 | 60 |
| Whakarewarewa Village | WHV18 | 2.5 | *Methylacidiphilum* | mNMS, V4 | 2.5 | 50 |
| Waikite Valley | WKT45* | 8.3 | *Methylacidiphilum* | mNMS, ANMS | 8.0 | 70 |
| Wairakei Thermal Valley | WTV1 | 4 | *Methylacidiphilum* | mNMS, V4 | 4.0 | 70 |
| Wairakei Thermal Valley | WTV2* | 7.7 | *Methylothermus, Methylacidiphilum* | mNMS, mmj | 8.0 | 75 |
| Wairakei Thermal Valley | WTV4 | 4 | *Methylacidiphilum* | mNMS, V4 | 4.0 | 60 |

Supplementary Materials 1. Supplementary methods.

- 1. Rationale for selection of growth media

Microcosms displaying > 0.01 % 16S rRNA gene sequence abundance and identified as *Methylococcus* (**Supplementary Table 2**) were inoculated into NMS media (Whittenbury et al., 1970), which has also been used to culture *Methylobacter* and *Methylosinus* strains (Whittenbury et al., 1970, Kalyuzhnaya et al., 2008). Microcosms with > 0.01 % of sequences assigned to *Methylomonas* were inoculated into dNMS (Dunfield et al., 2003, Hoefman et al., 2014), which can also support the growth of *Methylocystis*, *Methylocapsa* and *Methylocella* spp. (Dunfield et al., 2003, Dedysh et al., 2007, Dedysh et al., 2015). Neutral and alkaline pH microcosms (pH > 5.5), with > 0.01 % of 16S rRNA gene sequences identified as *Methylothermus*, were inoculated into mmj medium (Hirayama et al., 2011). This medium was previously used to isolate *Methylothermus subterraneus*, with which the 16S rRNA gene sequences from the community analysis in this study were most closely aligned. Five of these microcosms were additionally inoculated into ANMS medium, which has previously been used to isolate *Methylothermus thermalis* (Tsubota et al., 2005).

Microcosms at pH < 5.5 in which > 0.01 % of 16S rRNA gene sequences were identified as *Methylacidiphilum* spp. (**Supplementary Table 2**) were inoculated into V4 medium (Dunfield et al., 2007). V4 medium has been used to successfully cultivate acidophilic *Methylacidiphilum* and *Methylacidimicrobium* species (Sharp et al., 2012, Sharp et al., 2014). In microcosms OKO2 and TKA16 the only methanotrophs identified through 16S rRNA gene sequencing were *Methylacidiphilum*, but these represented only a very small proportion of the total reads (0.009 % and 0.005 %, respectively). As these microcosms were < pH 5.5 (pH 4.3 and 5.0, respectively), they were also inoculated into V4 medium. Fourteen microcosms that displayed > 0.01 % abundance of *Methylacidiphilum* sequences but a pH > 5.5 were not inoculated into V4 medium, as the maximum known growth pH for strains in this genus is pH 6.0 (Dunfield et al., 2007). Despite exhibiting methane oxidation, six of these microcosms (TOK15, WAP11, WHV13, WHV15, WHV16, WKT45) had no other methanotroph species detected via 16S rRNA gene amplicon sequencing. These microcosms were inoculated in ANMS medium (Tsubota et al., 2005) as a general methanotroph medium.

- 1. Culture media

**Modified NMS medium**: NaNO_3_, 0.084 g; MgSO_4_.7H_2_O, 0.135 g; CaCl_2_.6H_2_O, 0.026 g; KCl, 0.035 g; K_2_HPO_4_*OR* KH_2_PO_4_, 0.029 g; copper rich trace metal solution, 1 ml; ddH_2_O to 1 l. pH adjusted to that of sample.

**NMS medium**: MgSO_4_.7H_2_O, 1 g; KNO_3_, 1 g; CaCl_2_.6H_2_O, 0.2 g; Yeast extract, 0.01 g; NiCl_2_.6H_2_O, trace; NMS trace element solution, 1 ml; FeEDTA solution, 10 ml; ddH_2_O to 1 l. NMS phosphate buffer solution (added after autoclaving), 2 ml. pH 6.8

**dNMS medium**: Na_2_HPO_4_.12H_2_O, 0.4 g; MgSO_4_.7H_2_O, 0.2 g; KNO_3_, 0.2 g; CaCl_2_.6H_2_O, 0.04 g; Methanotrophs trace metal solution, 0.2 ml; FeEDTA solution, 0.2 ml; ddH_2_O to 1 l. pH 6.8

**ANMS medium:** KNO_3_, 0.25 g; NH_4_Cl, 0.25 g; KH_2_PO_4_, 0.13 g; Na_2_HPO_4_.12H_2_O, 0.358 g; MgSO_4_.7H_2_O, 0.4 g; CaCl_2_.2H_2_O, 0.1 g; Na_2_MoO_4_.6H_2_O, trace; NiCl_2_.6H_2_O, trace; B-Forte vitamin capsule, 0.01 g; Methanotrophs trace metal solution, 0.5 ml; FeEDTA solution, 0.2 ml; ddH_2_O to 1 l. pH 6.8

**mmj medium**: NaCl, 3 g; NaHCO_3_, 0.5 g; MgCl_2_.6H_2_O, 0.42 g; MgSO_4_.7H_2_O, 0.34 g; NH_4_Cl, 0.25 g; NaNO_3_, 0.25 g; CaCl_2_, 80 mg; KCl, 33 mg; K_2_HPO_4_, 14 mg; CuSO_4_, 3.7 mg; Fe(NH_4_)_2_(SO_4_)_2_.6H_2_O, 2 mg; B-Forte vitamin capsule, 0.01 g; Wolin trace mineral solution, 1 ml; ddH_2_O to 1 l. pH 6.2

**V4 medium**: NH_4_Cl, 0.4 g; KH_2_PO_4_, 0.05 g; MgSO_4_.7H_2_O, 0.02 g; CaCl_2_.6H_2_O, 0.01 g; Methanotrophs trace metal solution, 3 ml; FeEDTA solution, 3 ml; Wolin trace mineral solution, 1 ml; 1 mM Ce(SO_4_)_2_, 0.2 ml; 1 mM La_2_(SO_4_)_3_, 0.2 ml; ddH_2_O to 1 l. pH 2.5

**Copper rich trace metal solution**: CuSO_4_.5H_2_O, 4.1 g; ZnSO_4_.7H_2_O, 0.327 g; FeSO_4_.7H_2_O, 0.268 g; MnCl_2_.4H_2_O, 0.116 g; CoCl_2_.6H_2_O, 0.043 g; Na_2_MoO4.2H_2_O, 0.027 g; NiCl_2_.6H_2_O, 0.012 g; H_3_BO_3_, 0.008 g; Na_2_-EDTA, 2.032 g, ddH_2_O to 100 ml.

**NMS trace element solution**: CuSO_4_.5H_2_O, 200 mg; ZnSO_4_.7H_2_O, 10 mg; MnCl_2_.4H_2_O, 3 mg; H_3_BO_3_, 30 mg; CoCl_2_.6H_2_O, 10 mg; Na_2_MoO_4_.7H_2_O, 10 mg; ddH_2_O to 1 l. pH 4.0

**Methanotrophs trace metal solution:** ZnSO_4_.7H_2_O, 0.44 g; Na_2_MoO_4_.2H_2_O, 0.06 g; MnCl_4_H_2_O, 0.19 g; CuSO_4_.5H_2_O, 0.2 g; H_3_BO_3_, 0.10 g; CoCl_2_.6H_2_O, 0.08 g; ddH_2_O to 1 l.

**Clinicians B-Forte vitamin capsules** (Douglas Pharmaceuticals, Auckland, NZ) comprise: Choline bitartrate, 182 mg; Ascorbic acid, 100 mg; Thiamin hydrochloride, 84 mg; Riboflavin, 75 mg; Inositol, 75 mg; Nicotinamide, 75 mg; Pyridoxine hyrdrochloride, 61 mg; Calcium pantothenate, 55 mg; Folic Acid, 0.3 mg; Biotin, 0.1 mg; Cyanocobalamin, 0.05 mg.

Wolin trace metal solution: Nitrilotriacetic acid, 1.5 g; Fe(NH_4_)_2_(SO_4_)_2_·6H_2_O, 0.2 g; Na_2_SeO_4_.10H_2_O, 0.44 g; CoCl_2_·6H_2_O, 0.1 g; MnSO_4_·4H_2_O, 0.12 g; Na_2_MoO_4_·2H_2_O, 0.1 g; Na­WO_4_·2H_2_O, 0.1 g; ZnSO_4_·7H_2_O, 0.1 g; AlCl_3_·6H_2_O, 0.04 g; NiCl_2_·6H_2_O, 0.025 g; H_3_BO_3_, 0.01 g; CuSO_4_·5H_2_O, 0.01 g; ddH_2_O to 1 l.

**FeEDTA solution:** FeSO_4_.7H_2_O, 1.54 g; Na_2_-EDTA, 2.06 g, dd H_2_O to 1 l.

**NMS phosphate buffer solution:** Na_2_HPO_4_.12H_2_O, 35.8 g; KH_2_PO_4_, 13.6 g; ddH_2_O to 100 ml.

- 1. RNA extraction and sequencing

Cell pellets from metatranscriptome enrichments were resuspended in RNAlater before being sent to Novogene for RNA extraction and sequencing as per (Liu et al., 2015).

Total RNA was extracted using RNAiso Plus (TaKaRa Biotech. Co.)and treated with DNase I (TaKaRa Biotech. Co.). RNA quality, purity and quantity were checked using 1 % agarose gels, the RNA Nano 6000 Assay Kit of the Bioanalyzer 2100 system (Agilent Technologies), the Nano-Photometer® spectrophotometer (IMPLEN), and Qubit® RNA Assay Kit in Qubit® 2.0 Flurometer (Life Technologies).

First-strand cDNA was prepared by All-in-one First strand cDNA Synthesis Kit (Genecopoeia). qRT-PCR was performed using a BIO-RAD CFX96 q-PCR system with SYBR Green I fluorescent dye detection. The mRNA abundance was normalized with the housekeeping gene β-actin.

A total of 3 μg RNA per sample was used as input material for sequencing libraries generated using NEBNext® Ultra™ RNA Library Prep Kit for Illumina® (NEB) and index codes were added to attribute sequences to each sample. mRNA was purified from total RNA using poly-T oligo-attached magnetic beads. Fragmentation was carried out using divalent cations under elevated temperature in NEBNext First Strand Synthesis Reaction Buffer(5X). First strand cDNA was synthesized using random hexamer primer and M-MuLV Reverse Transcriptase(RNase H). Second strand cDNA synthesis was subsequently performed using DNA Polymerase I and RNase H. Remaining overhangs were converted into blunt ends via exonuclease/polymerase activities. After adenylation of 3’ ends of DNA fragments, NEBNext Adaptor with hairpin loop structure were ligated to prepare for hybridization. Library fragments were purified with AMPure XP system (Beckman Coulter) to preferentially 250 select cDNA fragments of ~ 300 bp in length Size-selected, adaptor-ligated cDNA was incubated with 3 μl USER Enzyme (NEB) at 37 °C for 15 min followed by 5 min at 95 °C before PCR. PCR was performed with Phusion High-Fidelity DNA polymerase, Universal PCR primers and Index (X) Primer. PCR products were purified (AMPure XP system) and quality assessed using the Agilent Bioanalyzer 2100 system. Library preparations were sequenced on a NovaSeq SP platform and 250 bp paired-end reads were generated.

Supplementary Table 3. Abundance of methanotroph-associated 16S rRNA gene sequences in GDS1 and TOK7 enrichment microcosms, and culture conditions for metatranscriptomic analysis.

|  |  | **Enrichment Microcosm** | |
| --- | --- | --- | --- |
|  |  | GDS1 | TOK7 |
| Incubation conditions | |  |  |
|  | Growth media | NMS | ANMS |
|  | Incubation temperature | 37 °C | 46 °C |
|  | pH | 7.2 | 6.8 |
|  |  |  |  |
| Abundance of known methanotrophs | |  |  |
|  | *Methylococcaceae* spp. | 5.5 % | 0 % |
|  | *Methylothermaceae* spp. | 0 % | 11.2 % |
|  | *Methylacidiphilum* spp. | 8.7 % | 17.9 % |

Supplementary Table 4. Methane oxidation in enrichment microcosms.

All microcosms were inoculated into at least two media, and the medium that resulted in the greatest rate of methane oxidation (listed, if any) is highlighted in **bold**.

| **Microcosm ID** | **Microcosm CH_4_ oxidation rate (µmol g^-1^ d^-1^)** | **Media** | **Media pH** | **Incubation**  **temperature (°C)** | **Enrichment CH_4_ oxidation rate (µmol d^-1^)** |
| --- | --- | --- | --- | --- | --- |
| GDS1 | 1.35 | mNMS, **NMS**, dNMS | 6.8 | 50 | 24.49 |
| GDS2 | 1.89 | mNMS, **NMS**, dNMS | 6.8 | 50 | 22.68 |
| LPR14 | 2.96 | mNMS, V4 | 3.0 | 75 | 0 |
| LPR16 | 0.57 | mNMS, **V4** | 1.5 | 70 | 4.56 |
| LPR17 | 1.00 | **mNMS**, V4 | 1.5 | 60 | 1.18 |
| NGM89 | 1.90 | **mNMS**, V4 | 3.0 | 60 | 5.54 |
| NGM91 | 1.63 | mNMS, **ANMS**, mmj | 6.8 | 60 | 10.64 |
| OKO2 | 17.36 | mNMS, V4 | 4.5 | 70 | 0 |
| TKA8 | 0.58 | **mNMS**, V4 | 5.0 | 75 | 1.32 |
| TKA9 | 2.50 | **mNMS**, V4 | 4.2 | 70 | 2.82 |
| TKA13 | 0.77 | **mNMS**, V4 | 4.0 | 70 | 5.18 |
| TKA15 | 0.67 | **mNMS**, V4 | 5.0 | 70 | 1.09 |
| TKA16 | 1.40 | **mNMS**, V4 | 5.0 | 70 | 1.82 |
| TKT67 | 9.35 | mNMS, V4 | 2.5 | 75 | 0 |
| TKT68 | 7.05 | mNMS, V4 | 3.0 | 37 | 0 |
| TOK7 | 4.40 | mNMS, mmj, **ANMS** | 6.8 | 60 | 3.48 |
| TOK10 | 2.72 | mNMS, mmj, **ANMS** | 6.8 | 60 | 7.67 |
| TOK12 | 1.51 | mNMS, mmj, **ANMS** | 8.0 | 60 | 8.88 |
| TOK15 | 0.58 | mNMS, ANMS | 6.0 | 70 | 0 |
| TOK17 | 5.48 | **mNMS**, mmj, ANMS | 7.5 | 60 | 5.98 |
| WAM36 | 1.67 | mNMS, V4 | 4.5 | 70 | 0 |
| WAP11 | N/A | mNMS, **ANMS** | 6.8 | 46 | 4.55 |
| WHV12 | 1.86 | **mNMS**, V4 | 3.0 | 60 | 6.54 |
| WHV13 | 0.48 | mNMS, ANMS | 8.5 | 75 | 0 |
| WHV15 | 0.68 | mNMS, ANMS | 6.0 | 75 | 0 |
| WHV16 | 1.53 | **mNMS**, ANMS | 5.5 | 60 | 0.47 |
| WHV18 | 1.84 | **mNMS**, V4 | 2.5 | 50 | 2.23 |
| WKT45 | 0.94 | mNMS, ANMS | 8.0 | 70 | 0 |
| WTV1 | 5.34 | **mNMS**, V4 | 4.0 | 70 | 2.29 |
| WTV2 | 1.40 | **mNMS**, mmj | 8.0 | 75 | 2.40 |
| WTV4 | 1.73 | mNMS, V4 | 4.0 | 60 | 0 |


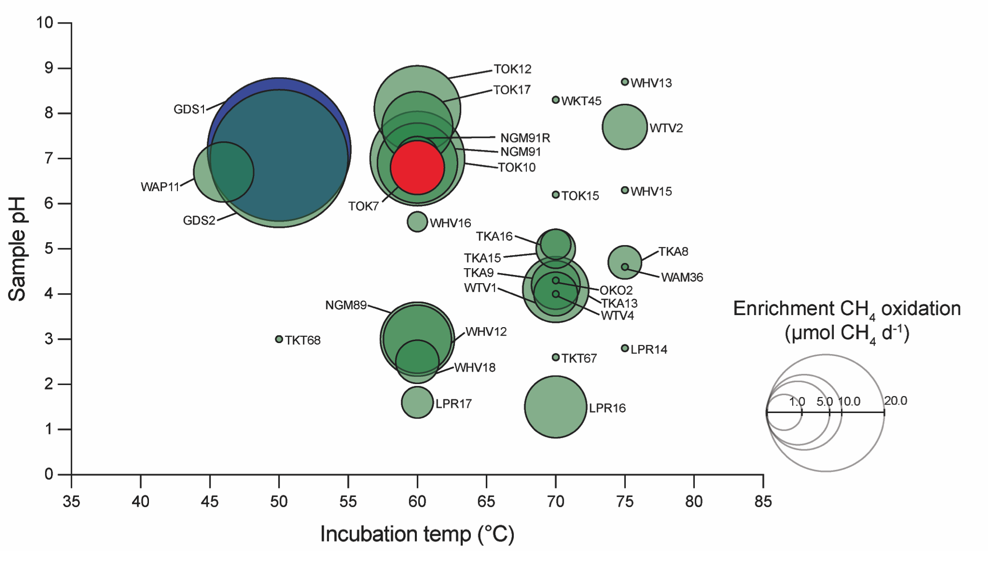


Supplementary Figure 4. Bubble charts illustrating observed methane oxidation rates of soil enrichments as a function of temperature and pH.

Green circles represent samples that oxidised methane and are labelled with an identifier. Bubble size is proportional to the rate of observed methane oxidation. Enrichments selected for metatranscriptome analysis, GDS1 and TOK7, are highlighted as blue and red respectively. COM; Craters of the Moon, GDS; Golden Springs, LPR; Loop Road, NGM; Ngatamariki. OKO; Orakei Korako; RTK; Rotokawa, TKA; Te Kopia, TKT; Tikitere, TOK; Tokaanu, WAM; Waimangu, WAP; Waipahihi; WHV; Whakarewarewa Village, WKT; Waikite Valley, WTV; Wairakei Thermal Valley.

GDS1 (37 °C)


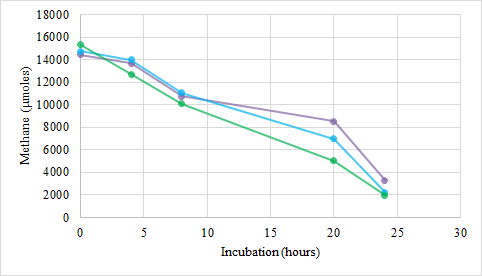


TOK7 (46 °C)


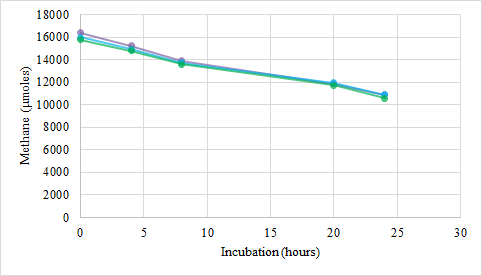


Supplementary Figure 5. Methane oxidation rates of samples selected for transcriptome analysis, before stabilisation and storage of RNA.

In both graphs, the three colours indicate each of the three biological replicates of the sample.

Supplementary Table 5. Most abundant transcripts from GDS1 biological replicates.

Transcripts that had an average transcripts per million (TPM) > 2000 across three biological replicates are shown. Identity is based on a BLASTx search using the NCBI non-redundant (nr) database.

| **Gene** | **TPM (avg)** | **% identity** | **e-value** | **Accession** | **Identity** | **BLASTx taxonomy** |
| --- | --- | --- | --- | --- | --- | --- |
| 38756 | 115475 | 96.6 | 4.1E^-55^ | AAB49820 | particulate methane monoxygenase subunit | *Methylococcus capsulatus* str. Bath |
| 23273 | 47157 | 96.8 | 1.5E^-109^ | AAB49820 | particulate methane monoxygenase subunit | *Methylococcus capsulatus* str. Bath |
| 23272 | 19414 | 99.2 | 2.5E^-180^ | AAB49821 | particulate methane monooxygenase 27 kDa subunit | *Methylococcus capsulatus* str. Bath |
| 23271 | 15301 | 71.3 | 0 | BAE86886 | methane monooxygenase protein B | *Methylomicrobium japanense* |
| 15955 | 11251 | 97.0 | 4.0E^-35^ | WP016920892 | hypothetical protein | *Methylocystis parvus* |
| 4623 | 6858 | 96.0 | 3.0E^-07^ | BAW80770 | hypothetical protein | *Candidatus* Nitrosoglobus terrae |
| 32715 | 5049 | 100 | 3.0E^-41^ | WP010960118 | cold-shock protein | *Methylococcus capsulatus* |
| 12629 | 4505 | 59.5 | 1.6E^-07^ | WP014889687 | methane monooxygenase/ammonia monooxygenase subunit C | *Methylocystis sp.* SC2 |
| 36297 | 3897 | 89.0 | 2.0E^-51^ | WP010962291 | hypothetical protein | *Methylococcus capsulatus* |
| 34484 | 3621 | 81.7 | 5.3E^-39^ | WP005372675 | methanol dehydrogenase | *Methylomicrobium album* BG8 |
| 34534 | 3569 | 96.0 | 7.0E^-45^ | WP006229561 | hypothetical protein | *Methylococcus capsulatus* |
| 39473 | 3564 | 74.0 | 3.0E^-43^ | WP007231737 | prepilin-type cleavage/methylation domain-containing protein | *Methylococcus capsulatus* |
| 37442 | 3071 | 94.0 | 9.0E^-53^ | WP010959880 | HU family DNA-binding protein | *Methylococcus capsulatus* |
| 40243 | 2799 | 100.0 | 8.0E^-33^ | WP010961245 | acyl carrier protein | *Methylococcus capsulatus* |
| 33708 | 2672 | 66.0 | 9.0E^-03^ | WP016918745 | hypothetical protein | *Methylocystis parvus* |
| 30531 | 2605 | 100 | 5.0E^-38^ | WP010959751 | carbon storage regulator | *Methylococcus capsulatus* |
| 35752 | 2469 | 98.0 | 5.0E^-64^ | WP010961831 | cytochrome c-555 | *Methylococcus capsulatus* |
| 6744 | 2285 | 87.0 | 4.0E^-55^ | WP086102986 | RNA-binding protein | *Chitinophagaceae* bacterium IBVUCB2 |
| 12617 | 2190 | 69.0 | 3.2E^-92^ | WP014889687 | methane monooxygenase/ammonia monooxygenase subunit C | *Methylocystis sp.* SC2 |

A total of 30.0 Gb trimmed and quality checked reads from three biological replicates of GDS1 (10.0 ± 0.7 Gb/sample) were obtained. GDS1 transcriptomes were then co-assembled using Trinity (Grabherr et al., 2011) into 34,259 clusters, which were predicted to include 63,694 genes using heuristic models (Besemer and Borodovsky, 1999). Clustering at 90 % nucleotide sequence identity retained 46,563 genes, and BLASTx (Altschul et al., 1997) was then used to identify proteins with significant alignments to the translated predicted genes (e-value < 0.01), with predicted metabolic functions assigned to 78.6 % of these reads (36,599 proteins). Biological replicates displayed a high degree of consistency, with 71.9 % of transcripts identified within each of the three GDS1 transcriptomes.

Supplementary Table 6. Most abundant transcripts from TOK7 biological replicates.

Transcripts that had an average TPM (transcripts per million) > 2000 across three biological replicates are shown. Identity is based on a BLASTx search using the NCBI non-redundant (nr) database.

| **Gene** | **TPM (avg)** | **% identity** | **e-value** | **Accession** | **Identity** | **BLASTx taxonomy** |
| --- | --- | --- | --- | --- | --- | --- |
| 40193 | 44756.4 | 96.0 | 1.0E^-08^ | WP077731218 | pyrroloquinoline quinone precursor peptide PqqA | *Methylocaldum* |
| 39757 | 14696.4 | 86.0 | 2.0E^-41^ | KXJ40929 | RNA-binding protein | *Methylothermaceae* bacteria B42 |
| 39638 | 12183.9 | 98.0 | 3.0E^-20^ | KXJ41919 | carbon storage regulator | *Methylothermaceae* bacteria B42 |
| 22578 | 12164.6 | 82.0 | 3.0E^-46^ | WP022948003 | HU family DNA-binding protein | *Methylohalobius crimeensis* |
| 14796 | 7729.3 | 92.0 | 6.0E^-36^ | WP089399015 | cold-shock protein | *Noviherbaspirillum humi* |
| 51290 | 7158.6 | 72.0 | 1.0E^-38^ | WP022948549 | hypothetical protein | *Methylohalobius crimeensis* |
| 22535 | 6690.8 | 77.0 | 5.0E-^27^ | KXJ39747 | hypothetical protein AXA67_11835 | *Methylothermaceae* bacteria B42 |
| 38267 | 6023.0 | 93.0 | 5.0E^-124^ | BAJ21968 | 3-hexulose-6-phosphate synthase | *Methylothermus subterraneus* |
| 51498 | 5505.7 | 64.0 | 2.0E^-57^ | WP022947508 | Hsp20/alpha crystallin family protein | *Methylohalobius crimeensis* |
| 38266 | 5437.4 | 94.0 | 2.0E^-120^ | BAJ21969 | 6-phospho-3-hexuloisomerase | *Methylothermus subterraneus* |
| 22546 | 5195.5 | 96.0 | 1.0E^-31^ | WP045224233 | acyl carrier protein | *Methyloterricola oryzae* |
| 38287 | 4957.3 | 65.7 | 0 | BAE86886 | methane monooxygenase protein B | *Methylomicrobium japanense* |
| 38285 | 4316.5 | 78.7 | 1.4E^-147^ | AAD43965 | particulate methane monooxygenase | *Methylococcus capsulatus* str. Bath |
| 60236 | 3697.7 | 84.0 | 6.0E^-107^ | WP022948752 | superoxide dismutase | *Methylohalobius crimeensis* |
| 39613 | 3672.7 | 77.0 | 4.0E^-84^ | WP022950180 | peptidylprolyl isomerase | *Methylohalobius crimeensis* |
| 51216 | 3499.5 | 90.0 | 8.0E^-05^ | GAM74922 | ATP synthase alpha chain | *Vibrio* |
| 100785 | 3371.1 | 71.0 | 4.0E^-20^ | WP022947738 | DUF1631 domain-containing protein | *Methylohalobius crimeensis* |
| 23254 | 3266.9 | 79.3 | 3.0E^-137^ | AAB49821 | particulate methane monooxygenase | *Methylococcus capsulatus* str. Bath |
| 38286 | 3145.2 | 89.0 | 0 | WP026596301 | PQQ-dependent dehydrogenase, methanol/ethanol | *Methylohalobius crimeensis* |
| 22551 | 2793.3 | 69.0 | 2.0E^-30^ | WP090829651 | DUF2188 domain-containing protein | *Nitrosovibrio tenuis* |
| 39715 | 2465.9 | 63.8 | 3.6E^-25^ | WP014331580 | cold-shock protein | *Sinorhizobium* |
| 60009 | 2451.6 | 78.8 | 2.1E^-40^ | WP003095657 | RNA-binding protein Hfq | *Pseudomonas* |
| 40164 | 2433.1 | 66.7 | 9.8E^-55^ | WP001280776 | thiol reductase thioredoxin | *Pseudomonadota* |
| 39832 | 2364.9 | 87.0 | 1.0E^-132^ | WP022946976 | peroxiredoxin | *Methylohalobius crimeensis* |
| 1685 | 2350.1 | 83.3 | 3.9E^-02^ | EAS50136 | hypothetical protein SI859A1_01496 | *Aurantimonas manganoxydans* |
| 38430 | 2324.6 | 67.4 | 5.4E^-42^ | WP011422641 | co-chaperone GroES | *Anaeromyxobacter* |
| 2280 | 2320.5 | 86.0 | 8.0E^-77^ | WP010961620 | cytochrome c | *Methylococcus capsulatus* |
| 44435 | 2160.0 | 81.8 | 1.6E^-34^ | WP011311367 | cold-shock protein | *Thiobacillus denitrificans* |

A total of 31.7 Gb trimmed and quality checked reads from three biological replicates of TOK7 (10.6 ± 1.3 Gb/sample) were obtained. TOK7 samples were co-assembled into 80,528 clusters, yielding 108,009 predicted genes. BLASTx identified 64,412 proteins with significant alignments to the translated predicted genes and replicates displayed similar expression patterns, with 48.1 % of transcripts identified in all three TOK7 samples.

**Reference**

Altschul, S. F., Madden, T. L., Schäffer, A. A., Zhang, J., Zhang, Z., Miller, W. & Lipman, D. J. 1997. Gapped BLAST and PSI-BLAST: A new generation of protein database search programs. *Nucleic Acids Research,* 25**,** 3389-3402 <https://doi.org/10.1093/nar/25.17.3389>

Besemer, J. & Borodovsky, M. 1999. Heuristic approach to deriving models for gene finding. *Nucleic Acids Research,* 27**,** 3911-3920 <https://doi.org/10.1093/nar/27.19.3911>

Dedysh, S. N., Belova, S. E., Bodelier, P. L. E., Smirnova, K. V., Khmelenina, V. N., Chidthaisong, A., Trotsenko, Y. A., Liesack, W. & Dunfield, P. F. 2007. *Methylocystis heyeri* sp. nov., a novel type II methanotrophic bacterium possessing ‘signature’ fatty acids of type I methanotrophs. *International Journal of Systematic and Evolutionary Microbiology,* 57**,** 472-479 <https://doi.org/10.1099/ijs.0.64623-0>

Dedysh, S. N., Didriksen, A., Danilova, O. V., Belova, S. E., Liebner, S. & Svenning, M. M. 2015. *Methylocapsa palsarum* sp. nov., a methanotroph isolated from a sub-Arctic discontinuous permafrost ecosystem. *International Journal of Systematic and Evolutionary Microbiology,* 65**,** 3618-3624 <https://doi.org/10.1099/ijsem.0.000465>

Dunfield, P. F., Khmelenina, V. N., Suzina, N. E., Trotsenko, Y. A. & Dedysh, S. N. 2003. *Methylocella silvestris* sp. nov., a novel methanotroph isolated from an acidic forest cambisol. *International Journal of Systematic and Evolutionary Microbiology,* 53**,** 1231-1239 <https://doi.org/10.1099/ijs.0.02481-0>

Dunfield, P. F., Yuryev, A., Senin, P., Smirnova, A. V., Stott, M. B., Hou, S., Ly, B., Saw, J. H., Zhou, Z., Ren, Y., Wang, J., Mountain, B. W., Crowe, M. A., Weatherby, T. M., Bodelier, P. L. E., Liesack, W., Feng, L., Wang, L. & Alam, M. 2007. Methane oxidation by an extremely acidophilic bacterium of the phylum *Verrucomicrobia*. *Nature,* 450**,** 879-882 <https://doi.org/10.1038/nature06411>

Grabherr, M. G., Haas, B. J., Yassour, M., Levin, J. Z., Thompson, D. A., Amit, I., Adiconis, X., Fan, L., Raychowdhury, R., Zeng, Q., Chen, Z., Mauceli, E., Hacohen, N., Gnirke, A., Rhind, N., Di Palma, F., Birren, B. W., Nusbaum, C., Lindblad-Toh, K., Friedman, N. & Regev, A. 2011. Full-length transcriptome assembly from RNA-Seq data without a reference genome. *Nature Biotechnology,* 29**,** 644-652 <https://doi.org/10.1038/nbt.1883>

Hirayama, H., Suzuki, Y., Abe, M., Miyazaki, M., Makita, H., Inagaki, F., Uematsu, K. & Takai, K. 2011. *Methylothermus subterraneus* sp. nov., a moderately thermophilic methanotroph isolated from a terrestrial subsurface hot aquifer. *International Journal of Systematic and Evolutionary Microbiology,* 61**,** 2646-2653 <https://doi.org/10.1099/ijs.0.028092-0>

Hoefman, S., Heylen, K. & De Vos, P. 2014. *Methylomonas lenta* sp. nov., a methanotroph isolated from manure and a denitrification tank. *International Journal of Systematic and Evolutionary Microbiology,* 64**,** 1210-1217 <https://doi.org/10.1099/ijs.0.057794-0>

Kalyuzhnaya, M. G., Khmelenina, V. N., Eshinimaev, B. T., Sorokin, D. Y., Fuse, H., Lidstrom, M. E. & Trotsenko, Y. A. 2008. Classification of halo(alkali)philic and halo(alkali)tolerant methanotrophs provisionally assigned to the genera *Methylomicrobium* and *Methylobacter* and emended description of the genus *Methylomicrobium*. *International Journal of Systematic and Evolutionary Microbiology,* 58**,** 591-596 <https://doi.org/10.1099/ijs.0.65317-0>

Power, J. F., Carere, C. R., Lee, C. K., Wakerley, G. L. J., Evans, D. W., Button, M., White, D., Climo, M. D., Hinze, A. M., Morgan, X. C., McDonald, I. R., Cary, S. C. & Stott, M. B. 2018. Microbial biogeography of 925 geothermal springs in New Zealand. *Nature Communications,* 9 <https://doi.org/10.1038/s41467-018-05020-y>

Sharp, C. E., Smirnova, A. V., Graham, J. M., Stott, M. B., Khadka, R., Moore, T. R., Grasby, S. E., Strack, M. & Dunfield, P. F. 2014. Distribution and diversity of *Verrucomicrobia* methanotrophs in geothermal and acidic environments. *Environmental Microbiology,* 16**,** 1867-1878 <https://doi.org/10.1111/1462-2920.12454>

Sharp, C. E., Stott, M. B. & Dunfield, P. F. 2012. Detection of autotrophic verrucomicrobial methanotrophs in a geothermal environment using stable isotope probing. *Frontiers in Microbiology,* 3 <https://doi.org/10.3389/fmicb.2012.00303>

Tsubota, J., Eshinimaev, B. T., Khmelenina, V. N. & Trotsenko, Y. A. 2005. *Methylothermus thermalis* gen. nov., sp. nov., a novel moderately thermophilic obligate methanotroph from a hot spring in Japan. *International Journal of Systematic and Evolutionary Microbiology,* 55**,** 1877-1884 <https://doi.org/10.1099/ijs.0.63691-0>

Whittenbury, R., Davies, S. L. & Davey, J. F. 1970. Exospores and cysts formed by methane-utilizing bacteria. *Journal of General Microbiology,* 61**,** 219-226 doi:10.1099/00221287-61-2-219
